# Protection of Malian children from clinical malaria is associated with recognition of multiple antigens

**DOI:** 10.1186/s12936-015-0567-9

**Published:** 2015-02-05

**Authors:** Modibo Daou, Bourèma Kouriba, Nicolas Ouédraogo, Issa Diarra, Charles Arama, Yamoussa Keita, Sibiri Sissoko, Boucary Ouologuem, Seydou Arama, Teun Bousema, Ogobara K Doumbo, Robert W Sauerwein, Anja Scholzen

**Affiliations:** Department of Epidemiology of Parasitic Diseases, Malaria Research and Training Centre, University of Science, Techniques and Technologies of Bamako, Bamako, Mali; Department of Medical Microbiology, Radboud university medical center, Route 268, PO Box 9101, 6500 HB Nijmegen, The Netherlands; Centre National de Recherche et de Formation sur le Paludisme (CNRFP), Ouagadougou, Burkina Faso; Department of Infection and Immunity, London School of Hygiene and Tropical Medicine, London, UK

**Keywords:** Malaria, *Plasmodium falciparum*, Antibody, Clinical protection, Stability

## Abstract

**Background:**

Naturally acquired immunity to clinical malaria is thought to be mainly antibody-mediated, but reports on antigen targets are contradictory. Recognition of multiple antigens may be crucial for protection. In this study, the magnitude of antibody responses and their temporal stability was assessed for a panel of malaria antigens in relation to protection against clinical *Plasmodium falciparum* malaria.

**Methods:**

Malian children aged two to 14 years were enrolled in a longitudinal study and followed up by passive and active case detection for seven months. Plasma was collected at enrolment and at the beginning, in the middle and after the end of the transmission season. Antibody titres to the *P. falciparum*-antigens apical membrane protein (AMA)-1, merozoite surface protein (MSP)-1_19_, MSP-3, glutamine-rich protein (GLURP-R0) and circumsporozoite antigen (CSP) were assessed by enzyme-linked immunosorbent assay (ELISA) for 99 children with plasma available at all time points. Parasite carriage was determined by microscopy and nested PCR.

**Results:**

Antibody titres to all antigens, except MSP-1_19_, and the number of antigens recognized increased with age. After malaria exposure, antibody titres increased in children that had low titres at baseline, but decreased in those with high baseline responses. No significant differences were found between antibody titers for individual antigens between children remaining symptomatic or asymptomatic after exposure, after adjustment for age. Instead, children remaining asymptomatic following parasite exposure had a broader repertoire of antigen recognition.

**Conclusions:**

The present study provides immune-epidemiological evidence from a limited cohort of Malian children that strong recognition of multiple antigens, rather than antibody titres for individual antigens, is associated with protection from clinical malaria.

**Electronic supplementary material:**

The online version of this article (doi:10.1186/s12936-015-0567-9) contains supplementary material, which is available to authorized users.

## Background

Despite the success of major public health control efforts in recent years [1], malaria remains one of the most important causes of morbidity and mortality in the world with an estimated 207 million cases and 627,000 deaths in 2012 [2]. Antibody-mediated immune responses to malaria antigens help to control blood-stage parasitaemia and have a protective effect on clinical disease, as shown in passive transfer experiments [3,4]. Identification of antigens that are the target(s) of these protective antibodies and their induction by natural exposure or immunization are a long-standing subject of fundamental and epidemiological studies as well as clinical vaccine trials [5].

The most commonly studied antigens in this respect are merozoite antigens, which are expressed both by mature liver schizonts as well as during the late schizont and merozoite-stage of blood-stage *Plasmodium* parasites [6]. Studies in different cohorts or transmission settings sometimes yield contradictory results [6]. One hypothesis is that a certain threshold of antibody responses has to be reached for them to be protective [7]. Other important considerations in immuno-epidemiological studies when assessing associations between immunological readouts, such as humoral responses and clinical protection, include the type of follow-up, the time point of assessment of humoral responses, the definition of ‘protection’ and the number of time points at which immunological readouts are assessed [6]. Additionally, recognition of multiple antigens in combination rather than just a single antigen are likely required for protection [8-10], which is already taken into account in several vaccine development studies and clinical trials [5].

Using a set of samples collected at four time points from a cohort of children followed up longitudinally for seven months, it was investigated whether a high concentration of antibodies against different pre-erythrocytic, blood-stage and cross-stage antigens, namely circumsporozoite antigen (CSP), merozoite surface protein (MSP)-3, apical membrane protein (AMA)-1, MSP-1_19_ and glutamine-rich protein (GLURP-R0), alone or in combination, may be associated with protection from clinical malaria in a hyper-endemic area. It was further assessed whether this association was temporally stable or dependent on the time point of sampling.

## Methods

### Study area

This study was conducted in Malian children from Samako, a village of about 3,000 people located in the Sudanese savannah zone of the Upper Niger valley (district of Kati) about 70 km southwest of Bamako, the capital of Mali. Samako is 5 km from Bancoumana where the Malaria Research and Training Centre has established a malaria vaccine site since 2000. *Plasmodium falciparum* is the predominant *Plasmodium* species in this region and accounts for more than 95% of malaria cases [11]. Transmission is mainly seasonal from June to December [11]. The study area has previously been described as a malaria hyper-endemic area [12]. From July to December of 2011, the overall incidence rate of clinical malaria during the transmission season period was 1.0 (244/243) episodes of malaria per person per season with 0.56 (28/50), 1.56 (78/50), 1.46 (102/70), 0.48 (35/73) episodes per person per season respectively in the age categories 3–11 months, 1–4 years, 5–14 years, and 15–50 years (Kone *et al.,* unpublished data).

### Ethical issues

Written informed consent was obtained from parents or legal guardians who consented on behalf of their children. All laboratory procedures were carried out within the guidelines of good laboratory practice. Ethical clearance to conduct the study was sought from the ethical committee of the Faculty of Medicine, Pharmacy and Odonto-Stomatology at the University of Science, Techniques and Technologies of Bamako (approval number 2011-58/FMPOS).

### Study subjects, design and conduct

One physician and one biologist were based in the village to follow up a cohort of children aged two to 14 years during one malaria transmission season. In December 2011, 171 children were enrolled in the study and subsequently attended three additional cross-sectional visits during the transmission season in July 2012 (n = 134) and September 2012 (n = 137) and after the end of the transmission season in February 2013 (n = 111). Ninety-nine children attended all three cross-sectional visits and provided samples during and after the 2012 transmission season and were selected for longitudinal immunological analysis in this study.

From July until December clinical malaria infection was monitored by active and passive case detection. The active case detection was carried out by house visits and cross-sectional survey. On a 14-day basis, field workers conducted active house visits to all the children to assess malaria infection. Participants were instructed to register any possible malaria symptoms in a diary. Passive case detection of clinical malaria episodes was carried out at the village health clinic. Children who visited the health clinic were identified using an individual identification card and were screened by a doctor. When children reported any of the malaria symptoms (fever, headache, diarrhoea, vomiting), a rapid diagnostic test (OptiMAL, Flow Inc, Portland, OR, USA) and thick smear were performed and axillary temperature was checked. A clinical malaria episode was defined by presence of any symptom of malaria including fever of 38°C or more, associated with a parasitaemia of at least 5,000 trophozoites/mm^3^, taking into account that low parasite densities are not always the causative factor of clinical symptoms [13]. Children with asymptomatic parasitaemia were not drug treated, neither at enrolment nor during follow-up.

**Figure 1:**
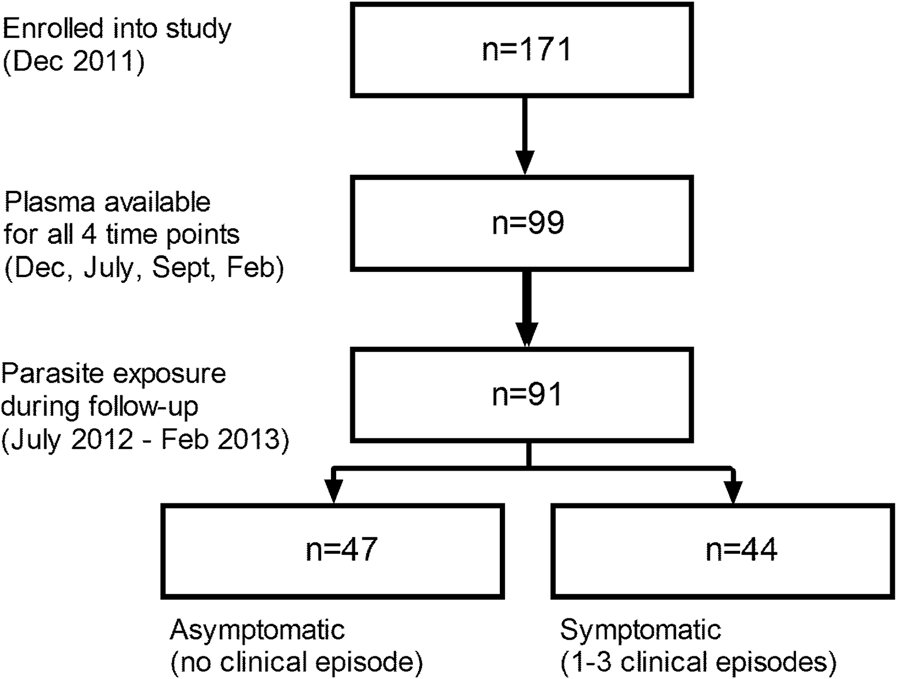
**Flow chart of subjects and sample availability for humoral analysis.** Of 170 children aged two to 14 enrolled in this study, 99 attended all three visits and provided plasma samples; 91 of these experienced parasitaemia (detected by thick smear or qPCR) during follow-up; 44 of these developed clinical disease once or more times.

Out of the 99 children providing plasma samples at all cross-section visits, 74 developed thick-smear detectable and 17 developed PCR-detectable infections with *P. falciparum* during follow-up (Figure 1). For eight children, no parasitaemia was detected at any time point during follow-up, so they were deemed unexposed. Children with co-infection of *P. falciparum* with other *Plasmodium* species, such as *Plasmodium malariae, Plasmodium ovale* or *Plasmodium vivax* were excluded from the analysis in the present study.

### Sample collection and sample processing

At each cross-sectional visit, 2–5 ml of blood was collected by venipuncture. About 20 μl of the blood was used to prepare a thick and thin smear for the detection of malaria parasites. Slides were stained with Giemsa at pH 7.2. A drop of whole blood was placed on Whatman filter paper strips number 3 (Whatman, Clifton, NJ, USA) and stored at room temperature for retrospective PCR analysis to detect sub-microscopic infections. EDTA anti-coagulated plasma was collected and cryopreserved at −20°C.

### Indirect enzyme-linked immunosorbent assay (ELISA)

The following antigens were used to assess humoral responses: (i) the ectodomain of apical membrane antigen (AMA)-1 of *P. falciparum* FVO, comprising amino acids 25–545, expressed in the yeast *Pichia pastoris* [14,15]; (ii) the conserved non-repeat N-terminal region R0 of glutamate rich protein (GLURP-R0; amino acids 85–213) [16]; (iii) full-length merozoite surface protein (MSP)-3 (K1) [17]; (iv) the 19-kilodalton carboxy-terminal fragment of MSP-1, expressed in *Escherichia coli* (MSP-1_19_) [18]; and, (v) full-length circumsporozoite protein (CSP) obtained from Genova Biotechniques Pvt. Ltd, Hyderabad, India.

While CSP is a pure pre-erythrocytic antigen expressed during the sporozoite and liver-stage of the parasite [19-21], and MSP-3 can only be detected on blood-stage parasites [22], AMA-1 [23-26], MSP-1 [27-29] and GLURP [30] are cross-stage antigens expressed during both the pre-erythrocytic and blood-stage of the parasite.

Microtitre plates (Maxisorb; NUNC) were coated with 50 μl of antigen diluted in PBS at a concentration of 1 μg/ml (AMA-1), 0.5 μg/ml (GLURP-R0), 0.18 μg/ml (MSP-1_19_), 0.5 μg/ml (MSP-3) or 0.25 μg/ml (CSP). Plates were incubated overnight at 4°C, washed and blocked with 200 μl of 5% milk powder in PBS for one hour. Due to the expected high inter-individual variation in antibody titers, plasma samples were assayed at two different dilutions (1:500 and 1:50) to ensure that at least one of the two would fit into the range of the standard curve. Fifty μl of diluted plasma samples were added in duplicate and incubated at room temperature for three hours. Plates were washed four times between steps. Polyclonal goat anti-human IgG-HRP (Pierce, Thermo Scientific) diluted 1:40,000 was added to antigen-coated plates. Bound secondary antibodies for total IgG were quantified with ready-to-use TMB (tetramethylbenzidine; TebuBio Laboratories) substrate for 30 min. Fifty μl of 0.2 M H_2_SO_4_ were used to stop the reaction. The plates were read out at the spectrophotometrical absorbance of 450 nm.

A plasma pool of hyperimmune Tanzanian adults living in a highly malaria-endemic area was used as serum standard, defined to contain 100 arbitrary units (AU) [31]. Optical density values for the test samples were converted into antibody units with the standard reference curves generated for each ELISA plate using a four-parameter, curve-fit, Microsoft Excel-based application ADAMSEL-v1.1 [32].

### Parasite detection by PCR

DNA was extracted from filter papers using the Chelex protocol as described by Walsh *et al.* [33]. The 18S PCR protocol developed by Snounou *et al.* [34] targeting the small ribosomal subunit of *P. falciparum* was used. PCR was performed according to the original protocol except that the quantity of template used in the N1 reaction was increased from 1 to 5 μl. In every set of PCR conditions 5 μL template was used in the N1 reaction and 1.5 μl of product in the N2 reaction. For a more detailed overview of primer sequences and product sizes, and PCR cycling conditions, see Additional file 1 and Additional file 2, respectively. Pooled DNA extracts from *P. falciparum* NF54 cultured in Nijmegen, The Netherlands were run on every PCR plate as a positive control, alongside a negative water control. The positive control was diluted to the extent that both N1 and N2 fragments were sufficiently amplified so that both amplicons could be visualized on agarose gel. N1 and N2 products were mixed and 10 μl was visualized on 0.8% agarose gel by electrophoresis in 0.5× Tris-acetate-EDTA buffer (0.04 M Tris-acetate and 1 mM EDTA, pH 8.0).

### Statistical analysis

Statistical analysis was performed using GraphPad Prism v5 software and STATA version 12.0 (Statacorp, College Station, Texas, USA). Differences in responses among different subject groups (different age groups; asymptomatic and symptomatic individuals; low, intermediate and high responders) were analysed by non-parametric measures. Mann–Whitney U test was used for two separate groups, Kruskal-Wallis with Dunn’s multiple comparison post-hoc test for more than two groups and Wilcoxon matched-pairs signed rank test for paired analysis of two time points. Associations between age and humoral responses were assessed by Spearman correlation; associations between the number of antigens recognized (AU > 10) and age in categories (2–5, 6–9 and 10–15 years), parasite status at enrolment (parasite-free, submicroscopic and microscopic parasite carriage) and ‘protection’ (asymptomatic parasite carriage or more than one clinical malaria episode) was determined by Poisson regression where the number of antigens recognized was analysed as count variable. Trends in the proportion of individuals recognizing ≥1 or ≥3 antigens and age in categories or parasite status were determined by logistic regression. The association between experiencing clinical malaria episodes or asymptomatic malaria and the density of antibody responses was further assessed by logistic regression models with antibody densities as categorical variables (<1 AU, 1–10 AU and ≥10 AU). For analyses where ELISA outcomes were analysed on a continuous scale, AU were log-transformed (log10) and analysed by linear regression. All associations were adjusted for age where appropriate. A p-value of < 0.05 was considered statistically significant.

## Results

### Association of antibody titres and breadth of the humoral response with age and parasitaemia

A longitudinal study was performed in 99 children during one malaria transmission season in Samako, a village in a hyperendemic area of Mali. The frequency of *P. falciparum* infections was highest during the July cross-sectional visit at the beginning of transmission season, with a frequency of slide positive children of 27.6% (27/98), and a proportion of parasite-positive children by PCR of 78.6% (77/98) (Table 1). Both the demographic and parasitological parameters of this longitudinal cohort were highly similar to the original total cohort of 171 children, from which this longitudinal cohort was selected based on attendance of all four cross-sectional visits (Additional file 3). The number of clinical malaria episodes recorded during longitudinal follow-up of this cohort from July to Dec 2012 peaked in October (July n = 1, Aug n = 8, Sept n = 9, Oct n = 19, Nov n = 17, Dec n = 4).

**Table 1:** **Demographic and parasitological parameters**

	**Dec 2011**	**Jul 2012**	**Sept 2012**	**Feb 2013**
Number of individuals (N)	n = 99			
Gender,% male (n/total)	55.6% (55/99)			
Age in year, median (range)	7 (2–14)			
Haemoglobin (g/dL), median (range)	11.6 (7.3-14.5)	11 (7.4-14.3)	11.6 (6.5-14.6)	12.2 (5.7-16.3)
Anaemia,% Hb <11 g/dL (n/total)	26.3% (26/99)	47.5% (47/99)	27.3% (27/99)	13.3% (13/98)^a^
Thick smear positive (%, n/total)	18.2% (18/99)	27.6% (27/98)^a^	20.2% (20/99)	10.8% (10/93)^a^
PCR positive (%, n/total)	n.d.	78.6% (77/98)^a^	43.6% (41/94)^a^	n.d.
Parasitaemia in thick-smear positive individuals (n = 35), median (range)	12,900 (100–51,300)	3,150 (100–132,275)	1,262 (100–58,675)	1,325 (100–7775)

n.d. not done.

^a^For some children not all data were collected at the time of visit.

**Figure 2:**
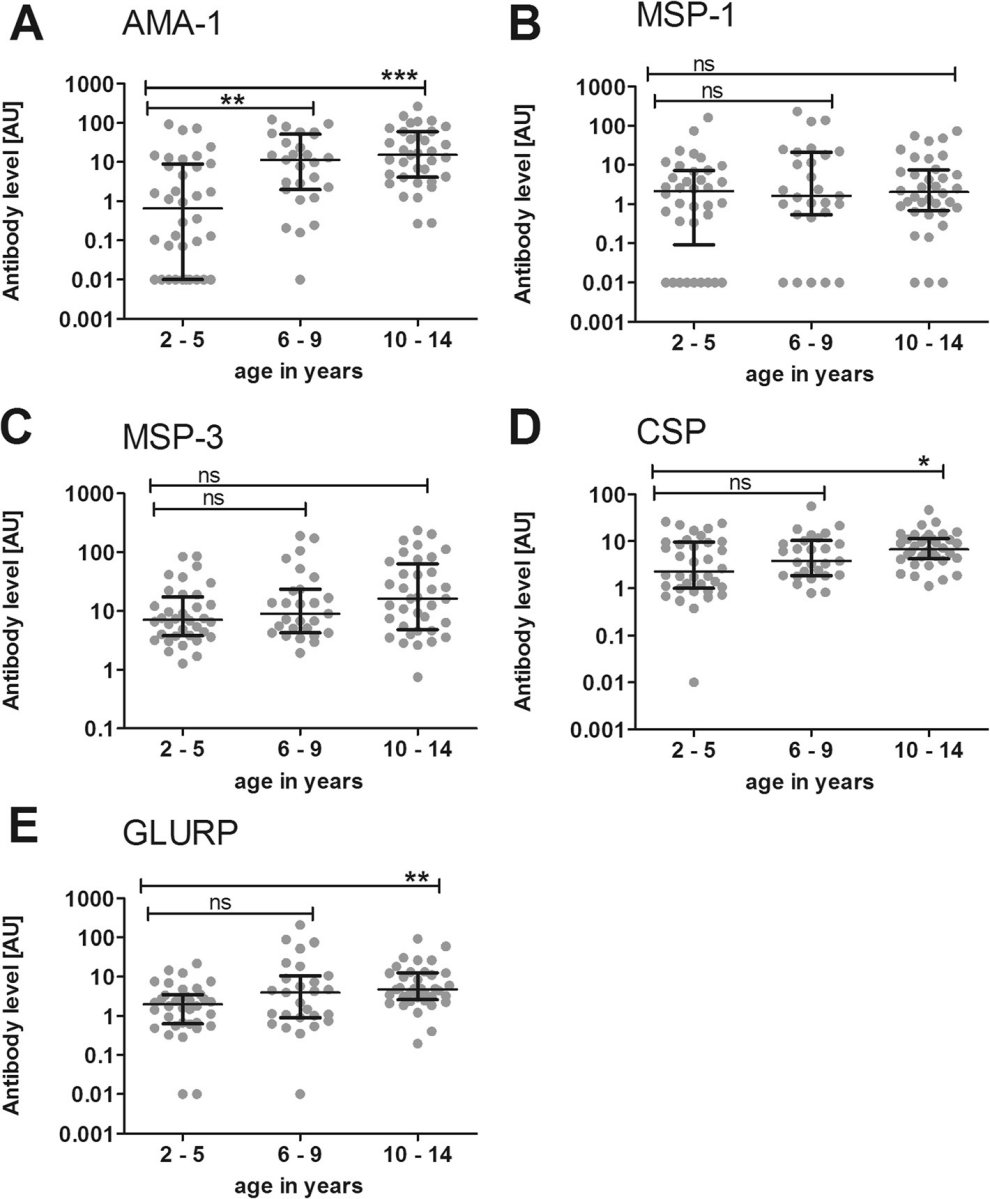
**Humoral responses by age at the beginning of the transmission season.** Antibody reactivity against *P. falciparum* antigens was assessed in samples from (n = 99) children collected at baseline. A pool of sera from 100 hyperimmune Tanzanians (HIT) was used as a standard positive control. Reactivity for each antigen in undiluted HIT serum was set at 100 arbitrary units (AU). Humoral reactivity was assessed against **(A)** AMA-1, **(B)** MSP-1_19_, **(C)** MSP-3, **(D)** CSP and **(E)** GLURP-R0. Children were divided into three different age groups: two to five years (n = 36), six to nine years (n = 27) and ten to 14 years (n = 36). Responses between the three age groups were compared using Kruskal-Wallis with Dunn’s multiple comparison post-test. *p < 0.05; **p < 0.01; ***p < 0.001. Scatter plots show individual data points, horizontal lines indicate the median of the group and error bars the interquartile range (IQR).

Antibody responses against five *P. falciparum* malaria antigens were assessed in the cohort at enrolment, the beginning of the transmission season (baseline), during and after the transmission season. At all time points there was a positive correlation between age and antibody titres for AMA-1, MSP-3, CSP and GLURP-R0, while responses to MSP-1_19_ were not associated with age (Additional file 4). Stratification of children into three age categories of two to five years, six to nine years and ten to 15 years revealed that the main increase in AMA-1 titres occurred before the age of six, while antibody levels for MSP-3, CSP and GLURP-R0 rose in a more continuous manner, which was evident both at baseline (Figure 2) and at the peak and end of the transmission season (Additional file 5 and Additional file 6).

**Figure 3:**
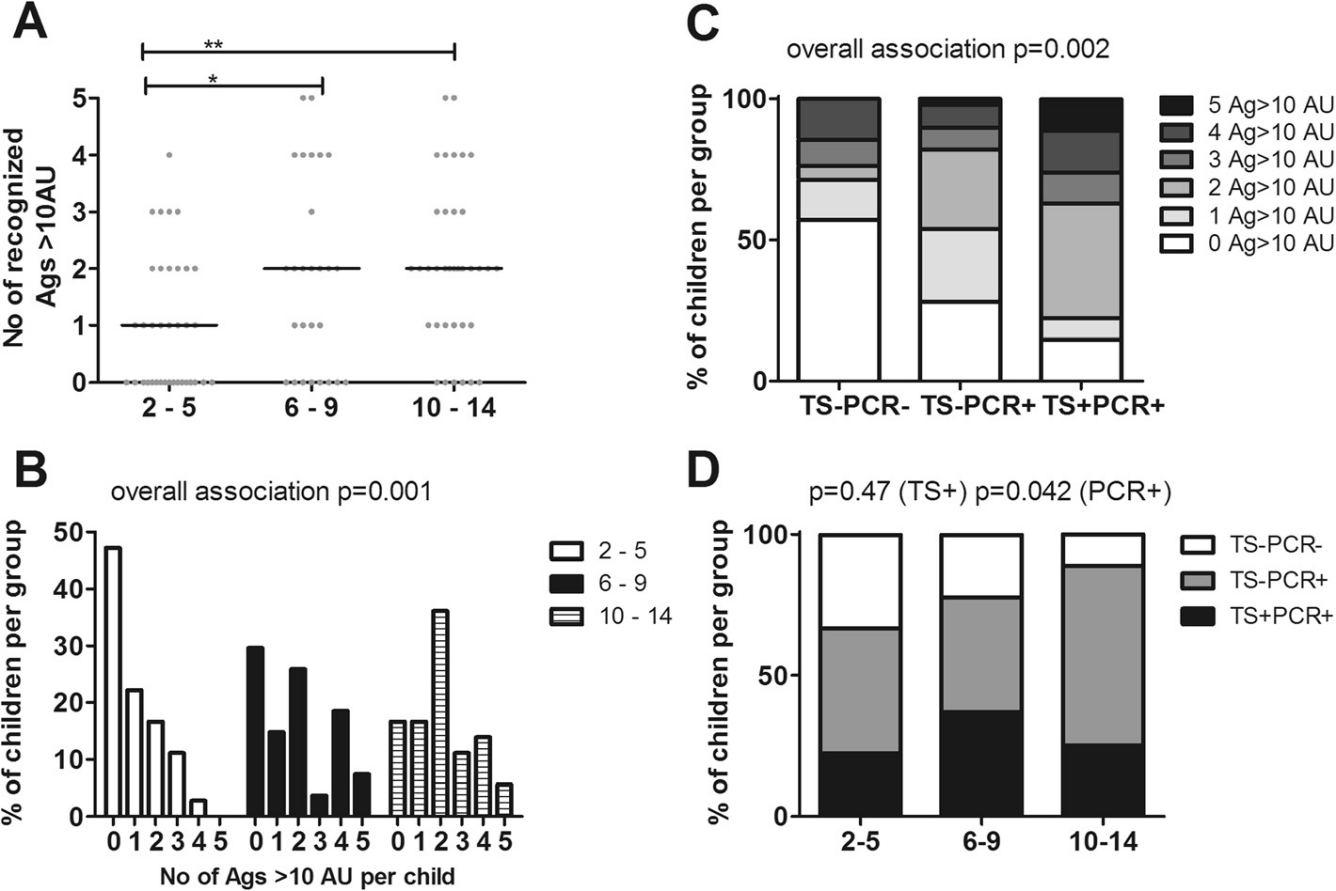
**Relationship of multiple antigen recognition with age and parasitaemia early in the transmission season.** The humoral response was analysed according to the number of antigens strongly recognized by each individual (n = 99) at the beginning of transmission season (July 2012). An arbitrary cut-off of 10 AU was used for each antigen and subjects were stratified by age. The three age groups: two to five years (n = 36), six to nine years (n = 27) and ten to 14 years (n = 36) were compared using Kruskal-Wallis with Dunn’s multiple comparison post-test. *p < 0.05; **p < 0.01; ***p < 0.001. **(A)** Scatter plots show individual data points, horizontal lines indicate the median of the group. **(B)** For each age group, the percentage of children strongly recognizing 0 or more antigens at baseline is shown and was analysed as count data by Poisson regression (overall association between age group and number of antigens recognized: p = 0.001). **(C)** Parasitaemia was assessed at the beginning of the transmission season (July 2012) by thick smear (TS) and PCR in 98 children (one TS negative child was excluded from analysis since no filter paper was available for PCR analysis). The proportion of children recognizing different numbers of antigens strongly (>10 AU) was stratified based on parasitaemia at time of sampling - not detectable (TS-PCR-; n = 21), sub-microscopic (TS-PCR+; n = 50) or microscopic (TS + PCR+; n = 27) and analysed as count data by Poisson regression (overall association between parasite status and the number of antigens recognized, p = 0.002). **(D)** The proportion of children with either no detectable (TS-PCR-; white), sub-microscopic (TS-PCR+; grey) or microscopic parasitaemia (TS + PCR+; black) in the three different age categories was analysed by logistical regression for the different diagnostics separately (p = 0.47 for TS+, p = 0.042 for total PCR+).

The next question was whether the breadth of the response also increased with age. Indeed, at all time points during follow-up there was a significant correlation between age and the number of antigens to which children showed an antibody level that reached at least 10% (>10 AU) of the hyperimmune reference serum (Additional file 4). Stratification into age groups showed that this increase occurred again before the age of six, with no difference between the older age groups (Figure 3A). Early in the transmission season (July 2012) 47.2% (17/36) of the children in the youngest age group had no high reactivity to any of the antigens, which decreased to 29.6% (8/27) in six to nine years old and 16.7% (6/36) in ten to 14 years old (p for trend = 0.007). While only 13.9% (5/36) of the two to five years old had high levels of antibodies against three or more antigens, this proportion was more than twice as high in six to nine year old children (29.6%, 8/27) and ten to 14 years old children (30.6%, 11/36); Figure 3B) (p for trend = 0.10). The breadth of the humoral response was significantly associated with parasitaemia at time of sampling (Figure 3C): 57.1% (12/21) of children with no detectable parasites recognized not a single antigen strongly, while this was only true for 28.0 (14/50) and 14.8% (4/27) of children with sub-microscopic or microscopic parasitaemia, respectively (p for trend = 0.007), after adjustment for age. While there was no significant difference in microscopically detectable parasitaemia between the different age categories (p = 0.79), PCR detectable parasitaemia, regardless of thick smear positivity, however, increased with age (p = 0.04; Figure 3D).

### Dependency of boosting of humoral responses on early season antibody levels

The breadth of the antibody response, i.e., the number of antigens strongly recognized (>10 AU) per individual, was stable over the time of follow-up, as evidenced by the strong correlation between this readout at enrolment (December 2011) and the beginning (July 2012; Spearman r = 0.59, p < 0.0001), peak (September 2012; r = 0.70, p < 0.0001) and end of the transmission season (February 2013; r = 0.67, p < 0.0001). Next it was investigated whether exposure during the transmission season altered antibody levels for the individual antigens. During the seven months of follow-up, 91/99 children became parasite-positive by either PCR or thick smear during one or more visits. Of these, 47 children remain asymptomatic while 44 experienced one or more episodes of clinical malaria (Figure 1).

Comparing antibody responses in all n = 91 malaria exposed children, there were no statistically significant difference for any of the antigens were observed between the beginning and end of the transmission season, (median AU [July 2012, February 2013]: AMA-1 [7.8, 7.2] p = 0.058; MSP-1_19_ [2.37, 2.51] p = 0.46; MSP-3 [11.6, 12.0] p = 0.27; CSP [6.14, 4.66] p = 0.18; GLURP-R0 [3.40, 3.09] p = 0.80). Since boosting of antibody responses may depend on the strength of the pre-existing response, all exposed children (n = 75) were stratified into low (<1 AU), intermediate (1–10 AU) and high responders (>10 AU) for each antigen. Low early season-responders for AMA-1, MSP-1_19_ and GLURP-R0 showed higher antibody levels after the transmission season, while these titers remained largely unchanged in intermediate early season-responders (Table 2). For MSP-3, there was only a single low early season-responder. For this antigen, intermediate early season-responders showed boosted antibody levels after the transmission season. The opposite was observed for children showing high early season antibody responses: for these children, post-transmission season antibody levels for MSP-1_19_, MSP-3 and CSP were significantly lower than their titers at the beginning of the season (Table 2). In contrast, boosting or waning of antibody titers was not depend on age, since without stratification by early season antibody titers, there was no significant difference between early and post-season antibody titers in any of the different age categories (Additional file 7).

**Table 2:** **Changes of antibody titers after parasite exposure during the transmission season dependent on baseline reactivity**

	**AMA-1**	**MSP-1**_**19**_	**MSP-3**	**CSP**	**GLURP-R0**
**<1 AU**	n = 21^a^	n = 28	n = 1	n = 6	n = 15
July 2012 median [IQR]	0.094 [0.01-0.25]	0.22 [0.01-0.6]		0.73 [0.50-0.86]	0.48 [0.2-0.62]
Feb 2013 median [IQR]	0.34 [0.09-2.0]	0.95 [0.05-2.0]		1.99 [1.39-2.24]	1.30 [0.88-2.16]
***p-value*** ^***b***^	***0.002***	***<0.0001***	***n.d.***	***0.03***	***0.002***
**1-10 AU**	n = 28	n = 39	n = 43	n =59	n = 56
July 2012 median [IQR]	3.0 [1.62-6.89]	2.5 [1.35-4.72]	5.05 [3.75-7.23]	4.16 [1.88-6.98]	3.15 [2.12-4.90]
Feb 2013 median [IQR]	4.4 [1.91-10.05]	2.46 [1.08-4.21]	7.21 [4.87-12.73]	4.66 [2.19-8.12]	3.06 [2.08-6.05]
***p-value*** ^***b***^	***0.067***	***0.96***	***<0.0001***	***0.11***	***0.33***
**>10 AU**	n = 42	n = 24	n = 47	n = 26	n = 20
July 2012 median [IQR]	37.1 [15.1-75.7]	23.9 [16.1-68.9]	29.8 [16.1-82.2]	14.7 [11.7-22.4]	22.4 [13.3-57.6]
Feb 2013 median [IQR]	36.1 [11.1-108.8]	15.3 [6.9-46.2]	19.9 [9.7-51.2]	8.1 [3.96-14.1]	20.2 [3.5-89.9]
***p-value*** ^***b***^	***0.45***	***0.047***	***0.002***	***<0.0001***	***0.59***

^a^91 children that experiences parasitaemia detected by qPCR or thick smear where included in this analysis.

^b^Antibody reactivity was compared between July and February for each group and antigen by Wilcoxon matched-pairs signed rank test.

Preferential boosting in the group of children with low antibodies at the beginning of the season could be due to the particularly high frequency of clinical malaria episodes during the transmission season in this group, compared to the intermediate or high early season-responders. This was indeed observed regardless of which antigen was used for stratification (Additional file 8). One possible explanation for lower antibody levels after the transmission season compared to baseline, as found in the high early season-responder group, might be temporarily elevated antibody levels at baseline due to an ongoing malaria infection. Indeed, there was a trend that the frequency of children with microscopic parasitaemia was highest in the early season high responder group and lowest in the early season low responder group for GLURP (p = 0.02), AMA-1 (p = 0.06) and MSP-1 (p = 0.11) (Additional file 9). For AMA-1 (p = 0.04) and MSP-3 (p = 0.03), this distinction was even found for the total proportion of children with any (either sub-microscopic or microscopic) parasitaemia, despite the fact that the majority of children (77/90) included in this longitudinal analysis was PCR positive at baseline.

### Association of antibody responses with clinical protection

**Figure 4:**
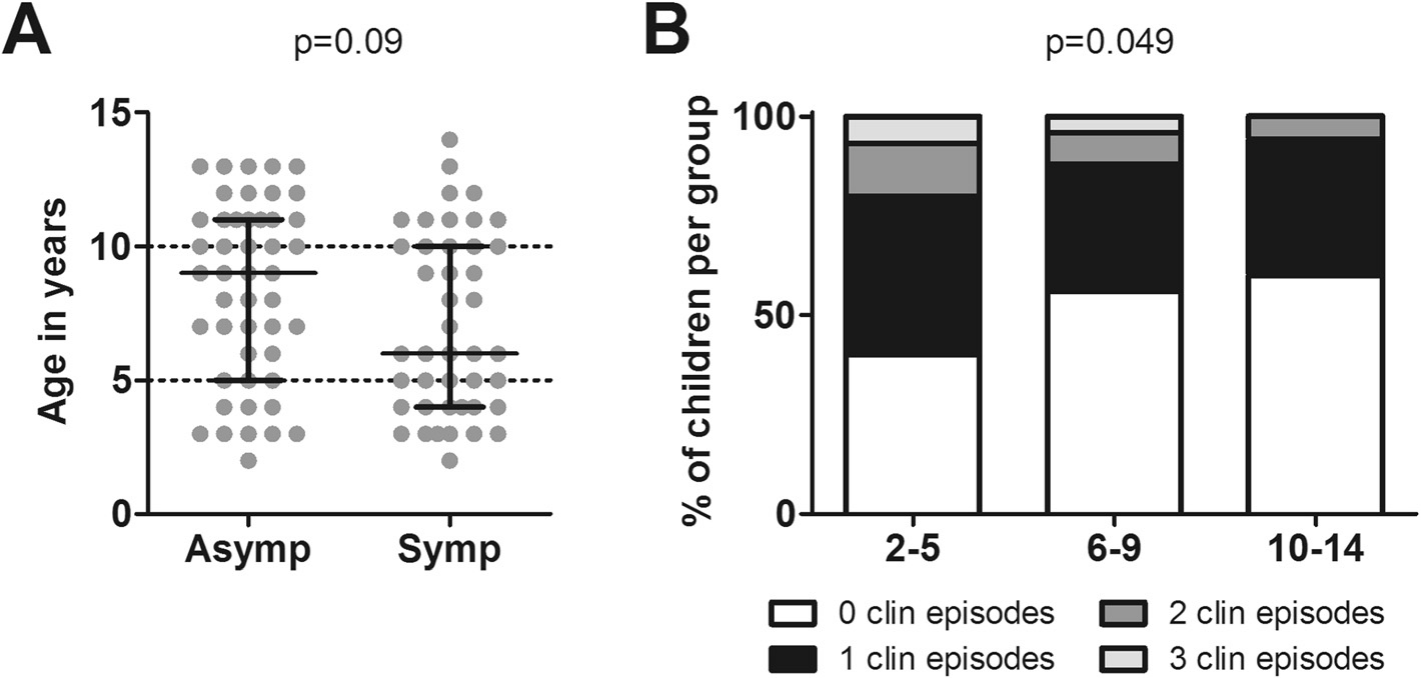
**Relationship between age and clinical protection. (A)** The age of all children who developed parasitaemia by PCR or thick smear during follow-up (total n = 91) in the asymptomatic (n = 47) and symptomatic groups (n = 44) is plotted. Scatter plots show individual data points, horizontal lines indicate the median of the group and error bars the interquartile range (IQR). The two groups were compared using Mann–Whitney U test. **(B)** The proportion of children experiencing 0, 1, 2 or 3 clinical episodes was calculated for the three different age groups (two to five years, six to nine years and ten to 14 years) and analysed as count data by Poisson analysis.

Because the youngest children showed overall weaker antibody responses and recognized a smaller number of antigens strongly than older children, it was next verified whether age might be a determinant for protection from clinical malaria during the transmission season. Overall, children remaining asymptomatic were older than those experiencing clinical malaria episodes, although this difference was not statistically significant (Figure 4A; p = 0.09). When age was dichotomized, children two to five years of age had a higher proportion of clinical episodes than the two older age groups (p = 0.049; Figure 4B). Therefore, all the following statistical analyses were adjusted for age.

**Figure 5:**
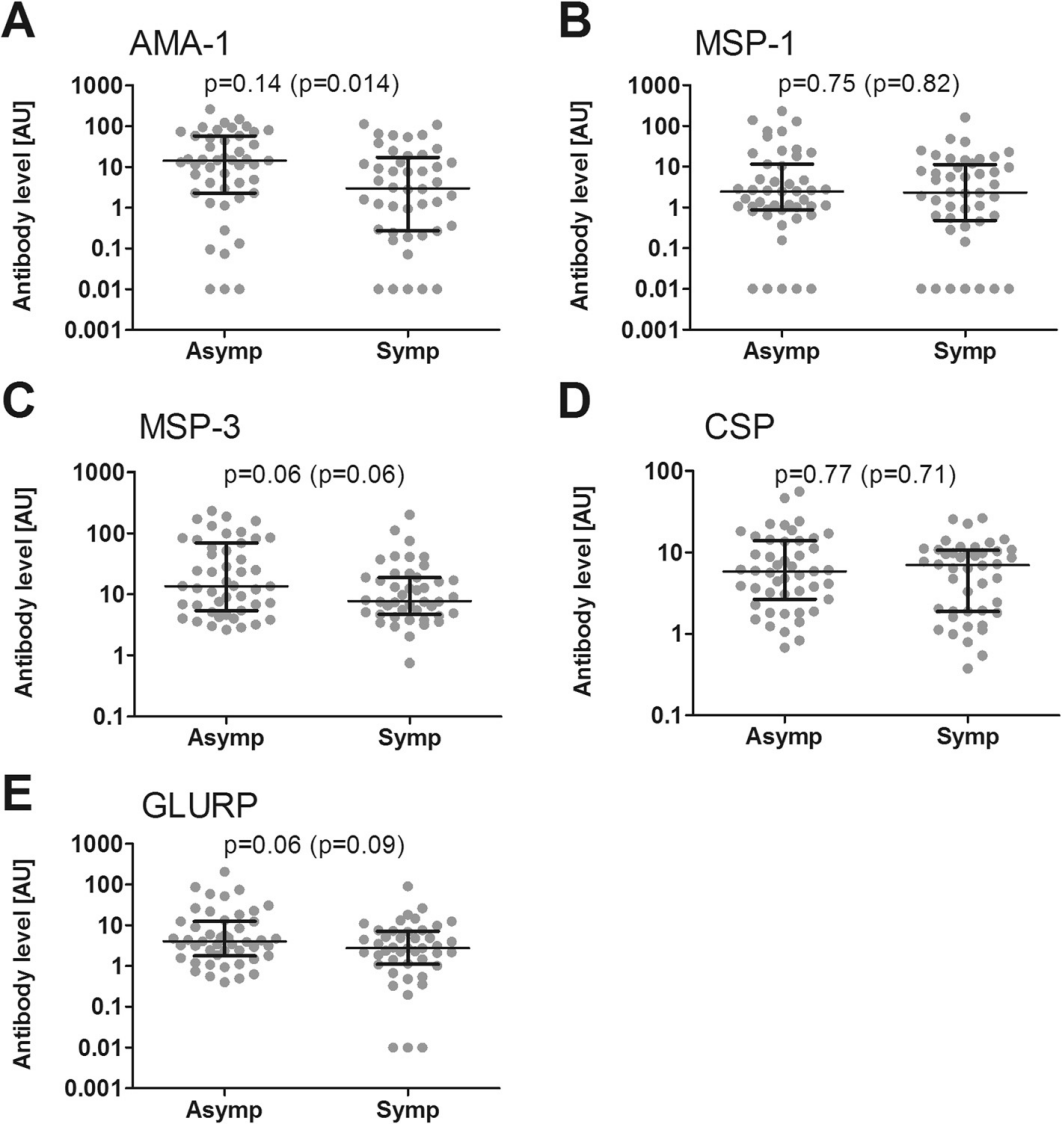
**Relation between antibodies at the beginning and clinical protection during the transmission season.** Humoral responses at the beginning of the transmission season were assessed by ELISA against **(A)** AMA-1, **(B)** MSP-1_19_, **(C)** MSP-3, **(D)** CSP and **(E)** GLURP-R0. The n = 91 children who developed parasitaemia by PCR or thick smear during follow-up were divided into two groups: a group consisting of asymptomatic children who experienced no clinical episode of malaria during the transmission season (n = 47), and a group of children who had a symptomatic malaria episode at least once during the transmission season (n = 44). Differences between the two groups were analysed by linear regression of log-transformed (log10) data, adjusting values for age. Age adjusted P values are shown for each plot, with p-values without age adjustment (Mann–Whitney U test) in brackets. Scatter plots show individual data points, horizontal lines indicate the median of the group and error bars the interquartile range (IQR).

Compared to children developing clinical malaria episodes, asymptomatic children showed significantly higher levels of antibodies against AMA-1, and non-significantly higher antibody responses for MSP-3 and GLURP-R0, at the start of the season (Figures 5A, C and E). These differences in antibody levels found early in the transmission season were also observed at enrolment and during longitudinal follow-up for AMA-1, MSP-3 and GLURP-R0 (Additional file 10A, C and E; Additional file 11A and C; Additional file 12A and E). However, none of these differences were statistically significant when adjusted for age. The levels of anti-MSP-1_19_ and anti-CSP antibodies were comparable between children remaining asymptomatic and symptomatic at enrollment (Additional file 10) and during the entire follow-up (Additional files 11B and 12B). When antibody responses were dichotomized as high responders (>10 AU) and low responders, only high antibody responses to AMA-1 (AU > 10) in July 2012 were associated with a lower risk of clinical malaria episodes in the subsequent season (OR 0.37, 95% CI 0.15-0.91, p = 0.03). No such association was observed MSP-1 (OR 0.92, 95% CI 0.35-2.37, p = 0.86), MSP-3 (OR 0.60, 95% CI 0.25-1.40, p = 0.24), GLURP (OR 0.62, 95% CI 0.21-1.80, p = 0.38) or CSP (OR 1.00, 95% CI 0.39-2.57, p = 0.99).

**Figure 6:**
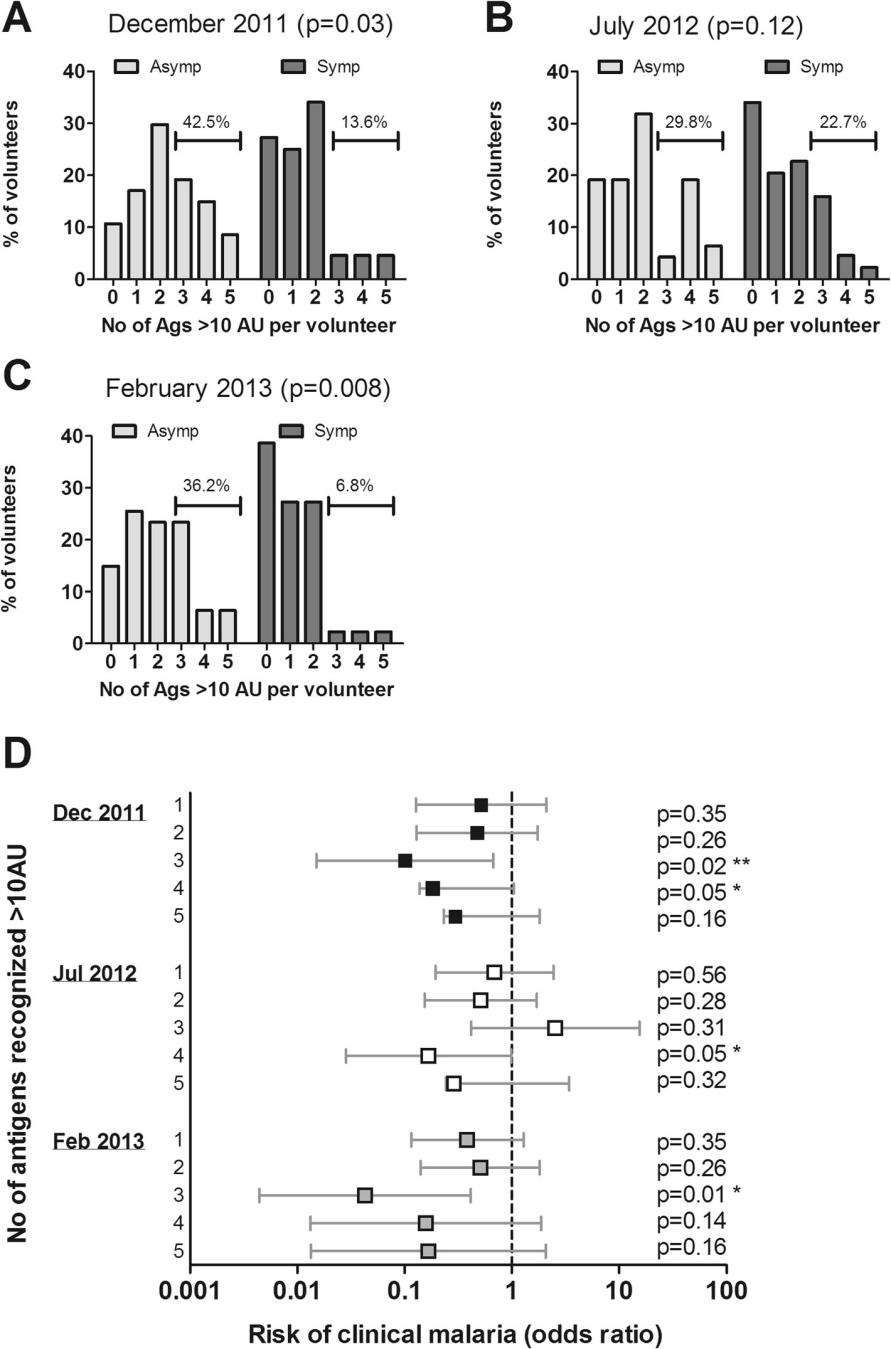
**Relationship between strong humoral responses to multiple antigens and risk of clinical malaria.** Children who developed parasitaemia by PCR or thick smear during follow-up (n = 91) were divided into asymptomatic children who experienced no clinical episode of malaria during the transmission season (n = 47), and children who had a symptomatic malaria episode at least once during the transmission season (n = 44). For both groups, the percentage of children strongly recognizing 0 or more antigens is shown at **(A)** enrollment, **(B)** in the beginning and **(C)** after the transmission season. An arbitrary cut-off of 10 AU was used for each antigen. Horizontal bars indicate the percentage of children in each group recognizing three or more antigens strongly. The number of antigens to which high reactivity (>10 AU) was observed was analysed as count data by Poisson regression. **(D)** The risk of developing clinical malaria during the transmission season was calculated by logistic regression analysis for children that were high responders (>10A AU) for 1, 2, 3, 4 or 5 antigens simultaneously in reference to those that recognized not a single antigen strongly, with adjustment for age. Analysis was performed either prospectively (using December 2011 or July 2012, i.e. before clinical episodes were recorded, excluding one volunteer that experienced a clinical episode in July from the analysis), or retrospectively (using post-season February 2013 antibody titers). Symbols depict odds ratios, error bars indicate the upper and lower 95% confidence interval.

While recognition of individual antigen was therefore no good predictor of protection from clinical disease, children remaining asymptomatic recognized a broader repertoire of antigens compared to those that developed clinical disease (Figure 6A (December 2011, p = 0.03); B (July 2012, p = 0.12); C (February 2013, p = 0.008)). Although not always statistically significant, across all time points analysed, a greater proportion of children that became symptomatic during the season lacked high reactivity to any of the antigens compared to asymptomatic children, while strong responses to three or more antigens were found in a greater proportion of asymptomatic children compared to those that were clinically unprotected (Figure 6). Amongst those children with high reactivity for three to five antigens, 95.4% had strong responses for MSP-3 (median across all time points), 81.0% for AMA-1, 72.3% for CSP, 66.3% for GLURP-R0 and 53.8% for MSP-1_19_. Children strongly recognizing three or more antigens has a reduced risk of developing clinical malaria, both when antibody titers were assessed prior to (prospective analysis, December 2011 and July 2012) or after (retrospective analysis, February 2013) the 2012 transmission season, in which clinical malaria was detected (Figure 6D). Again, these data did not all reach significance, but showed a clear trend across time points.

## Discussion

Immuno-epidemiological studies in multiple settings are needed to further an understanding of the relationship between naturally acquired antibody responses to the parasite and protection from clinical malaria. The key finding of the present study is that in a highly endemic area in Mali, the risk of developing clinical malaria upon exposure was reduced when children showed strong recognition of three or more malaria antigens at any time point during the study, regardless whether those responses were measured before or after the malaria risk was assessed.

Studies into antibody correlates of protection can be affected by a number of confounders. A meta-analysis of other studies assessing the association between antibodies and protection from clinical disease found that most studies do not exclude individuals without detected parasitaemia during follow-up from the ‘protected’ group, although there is no evidence that these individuals were even exposed [6]. Such practice may decrease the magnitude of the observed effect between high and low responders [35]. In the present study, individuals without evidence of malaria exposure during the study, as assessed by PCR and microscopy, were excluded from the analysis on clinical protection. In this context, a limitation of any immune-epidemiological study including the present one, in assessing exposure to the parasite is the frequency of monitoring for parasitaemia. Ideally, parasitaemia would be determined at weekly intervals by PCR. In the present study, parasitaemia was only assessed microscopically at the four cross-sectional visits, or when children reported malaria symptoms during the bi-weekly active case detection visits or at the local health clinic. Furthermore, PCR data were only available for two of the cross-sectional visits. This sampling approach detected many asymptomatic infections that allowed us to exclude potentially unexposed individuals [35], but may not have captured all infections and therefore may have resulted in the exclusion of some individuals with asymptomatic infections of short duration [36]. Of note, in the present study setting, a considerable proportion of infections were detectable by PCR only [37,38]. Parasite prevalence was almost threefold higher by PCR than by microscopy, which has been reported before but is higher than typically observed in areas with this level of transmission intensity [37]. The findings suggest that a considerable proportion of children are capable of controlling infections to densities below the microscopic threshold density for detection.

Age, reflecting cumulative malaria exposure, is an important determinant of protection against clinical malaria. In this study, children ranging from age two to 14 years were enrolled. A decreasing risk for clinical malaria [39], severe disease [40], and an increase of antibody responses [41,42] with age are commonly reported. Accordingly, in the present study, an increase in humoral responses and clinical protection particularly up to the age of six years was also observed. As a result, apparently stronger antibody responses to AMA-1, MSP-3 and GLURP-R0 at the beginning of the transmission season in children protected from clinical disease were found to be largely explained by their higher age. Nevertheless, even when adjusting for age, high responders to AMA-1 and high responders to a combination of three or more antigens had a lower risk for clinical disease. Therefore, it is no age *per se*, but the acquisition of specific and strong responses with the degree of cumulative exposure that determines humoral protection.

An important consideration when interpreting the findings from the current study is the size of the study cohort. In the current study, the number of children from which samples could be analysed was reduced by a number of factors, including commitment to longitudinal assessment and attendance of all cross-sectional visits. This approach was chosen to allow for a single consistent data set to be used for all analysis, as opposed to doing analysis for each time point with a different, only partially overlapping set of participants. While this did not lead to a skewing in the demographic or parasitological parameters compared to the original cohort, the two groups of children remaining asymptomatic or becoming symptomatic upon parasite exposure were too small for several of the results to reach statistical significance. Nevertheless, clear trends were observed throughout time points, justifying future studies in larger cohorts.

It was previously highlighted that studies measuring responses at multiple time points are needed to better determine the serological status of an individual and whether the time point of observation may have an impact on the association of the humoral responses with protection [6,43]. Changes in antibody titres after exposure were, therefore, also examined. Overall, breadth and magnitude of the antibody response in this cohort were stable over the period of observation that comprised one complete transmission season. This stability of antibody responses may explain why associations of clinical protection with recognition of multiple antigens could be observed at any time point examined. This may, however be different in other transmission settings, where antibody levels are less stable. With the exception of AMA-1, boosting of antibody titres during the transmission season could only be observed when children were stratified by their baseline antibody levels, rather than when analysed as a whole group or stratified by age. Children with low baseline antibodies level showed boosting of blood- and cross-stage antigens and a similar trend for the pre-erythrocytic antigen CSP. Surprisingly, children with high baseline antibodies level (>10 AU) showed decreased rather than increased titres after the transmission season, which coincided with a lower frequency of clinical malaria episodes that these children experienced during the transmission season. The risk of symptomatic malaria and disease severity is often associated with higher parasitaemia [39,44]. Whether the lower frequency of clinical malaria episodes in this group also reflects lower parasitaemia and hence less antigen exposure during asymptomatic episodes in the transmission season could not be determined, since parasitaemia measurements were available from too few time points. Lower antigen exposure would, however, at least partially explain the less efficient boosting of antibody responses in this group of children.

The apparent decrease in antibody titres over the transmission season in these children might be an artifact caused by asymptomatic parasitaemia at time of plasma sampling: At the beginning of the transmission season the group of children with the highest antibody titres for individual antigens also had the highest frequency of parasitaemia, detected either by thick smear or PCR.

That concurrent, even asymptomatic, low-density parasitaemia is associated with elevated antibody titres or prevalence, is in line with previous data [35,41,45-48]. Similarly, the number of strongly recognized antigens was also higher when children carried parasites at the time of sampling, again in line with other studies [10]. Such elevated antibody levels during or shortly after acute infection mostly stem from short-lived plasma blasts, only some of which will eventually establish themselves in survival niches as long-lived plasma cells [49]. Contraction of this short-lived response soon after parasite clearance results in a drop of antibody levels that is well reported for malaria antigens [47,50] and might be relatively greater than the small increase in stable antibody levels (in the absence of parasites or exposure) derived from long-lived plasma cells induced during the transmission season [42].

Amongst the four blood- and cross-stage antigens examined, MSP-1_19_ was the only one for which reactivity did not increase with age, nor did titers for it differ at all clinical protection in this study. Although antibodies to MSP-1_19_ are generally found to confer protection [6,51], this effect is weakest for the Wellcome allele of MSP-1_19_ [6] and also was not found by all studies [52], the reason for which could be the exact source and sequence of antigen, but also actual difference between cohorts. The result of the present study is in line with a previous study finding no protection-association when using the same MSP-1_19_ antigen [10]. Antibody titers for the other blood- and cross-stage antigens AMA-1, MSP-3 and GLURP showed either no significant difference or only trends for slightly higher responses in children remaining asymptomatic; any apparent differences here were clearly age-confounded. Further, there was no difference in CSP antibody titers between children protected or not from clinical disease. Although occasionally reported [53], CSP responses are not commonly found to be associated with clinical protection [54-57], but can rather confer protection from infection at high titres [58].

When analysed as single antigens, only antibody responses to AMA-1 were found to be associated with a reduced risk of clinical malaria. In contrast, high responses for a combination of at least 3/5 malaria antigens showed a relatively consistent protective effect across all time points analysed. These findings are in line with previous studies showing that protection against malaria may not be associated with the humoral responses to one antigen in isolation, but instead with recognition of a panel of antigens [8-10,59,60]. Moreover, MSP-3 and AMA-1 were more prevalently recognized than other antigens amongst children showing responses to multiple antigens, confirming a previous study showing that recognition of these two antigens in combination was more strongly associated with protection than other combinations [10]. In the present study, assessment of antibody responses was limited to five classical vaccine candidate antigens by conventional ELISA. Recently, it was reported that Kenyan children have antibody responses against novel and little-studied merozoites proteins, which had high protective efficacy, particularly when five out of the ten leading proteins were recognized [59]. Application of protein microarrays probed with a large set of *P. falciparum* antigens to samples from the present and similar studies with carefully defined, protected and non-protected cohorts will be a useful tool to extend these findings and add to the first few existing studies to identify novel antigens and panels thereof associated with protection from clinical disease [9,54]. Based on this, screening for a panel of antigens in field studies could be implemented using bead-based multiplex ELISAs [61-63], which are more cost-efficient than full protein microarrays.

## Conclusions

The present study provides immune-epidemiological evidence from a limited cohort of Malian children that protection from clinical malaria is associated with high antibody titres for three or more malaria antigens. Future studies should confirm these findings for other settings and study periods with study populations that are adequately powered to detect more subtle associations between antibody recognition, age and clinical malaria.

## Additional files

Additional file 1:
**Primer sequences and product sizes.** Provides details of primers used to detect *Plasmodium falciparum* DNA extracted from filter papers by PCR. Additional file 2:
**PCR Mastermix composition and PCR thermal cycler programme.** Provides details of methodology and PCR cycling conditions used to detect *Plasmodium falciparum* DNA extracted from filter papers by PCR. Additional file 3:
**Demographic and parasitological parameters of total cohort.** Provides demographic and parasitological data for all individuals that attended one or more of the four cross-sectional visits, out of which the longitudinal cohort of n = 99 children attending all visits was selected for immunological analysis. Additional file 4:
**Correlation between age and humoral responses during follow-up.** Spearman correlation analysis was performed to analyse for all n = 99 children the relationship between age and the height of antibody titers to the five malaria antigens AMA-1, MSP-1_19_, MSP-3, CSP and GLURP-R0 at each of the four visits. Additional file 5:
**Humoral responses by age in the middle of the transmission season.** Antibody reactivity against *P. falciparum* antigens was tested by ELISA on samples from (n = 99) children collected in the middle of transmission (September 2012). A pool of sera from 100 hyperimmune Tanzanians (HIT) was used as a standard positive control. Reactivity for each antigen in undiluted HIT serum was set at 100 arbitrary units (AU). Humoral reactivity was assessed against (A) AMA-1, (B) MSP-1_19_, (C) MSP-3, (D) CSP and (E) GLURP-R0. Responses between the three age groups were compared using Kruskal-Wallis with Dunn’s multiple comparison post-test. *p < 0.05; **p < 0.01; ***p < 0.001. Scatter plots show individual data points, horizontal lines indicate the median of the group and error bars the interquartile range (IQR). Additional file 6:
**Humoral responses by age after the transmission season.** Antibody reactivity against *P. falciparum* antigens was tested by ELISA on samples from (n = 99) children collected after the transmission (February 2013). A pool of sera from 100 hyperimmune Tanzanians (HIT) was used as a standard positive control. Reactivity for each antigen in undiluted HIT serum was set at 100 arbitrary units (AU). Humoral reactivity was assessed against (A) AMA-1, (B) MSP-1_19_, (C) MSP-3, (D) CSP and (E) GLURP-R0. Responses between the three age groups were compared using Kruskal-Wallis with Dunn’s multiple comparison post-test. *p < 0.05; **p < 0.01; ***p < 0.001. Scatter plots show individual data points, horizontal lines indicate the median of the group and error bars the interquartile range (IQR). Additional file 7:
**Changes of antibody titers after parasite exposure during the transmission season dependent on age.** All n = 91 children that were exposed to the malaria parasite during follow-up were divided into age categories of two to five years (n = 35), six to nine years (n = 27) and ten to 14 years (n = 36). Antibody titers early during (July 2012) and after the transmission season (February 2013) were compared by Wilcoxon matched-pairs signed rank test. Additional file 8:
**Frequency of clinical malaria episodes in children stratified based on their reactivity to different** ***Plasmodium falciparum*** **antigens.** All n = 91 children that were exposed to the malaria parasite during follow-up were stratified based on their antibody responses to individual malaria antigens in July 2012 (early in the transmission season) as follows: <1 AU, i.e., <1% of reference HIT serum; 1–10 AU; >10 AU, i.e., >10% of reference HIT serum. Additionally children were also stratified based on the number of antigens strongly recognized at this time point, using an arbitrary cut-off of 10 AU (0 antigens, 1–2 antigens and 3–5 antigens). The proportion of children developing clinical malaria in each group during follow-up is shown. Additional file 9:
**Degree of parasitaemia in groups with different humoral reactivity. **For each antigen, children were stratified into three groups depending on their response to this respective antigen early in the transmission season (July 2012) as follows: <1 AU, i.e., <1% of reference HIT serum; 1–10 AU; >10 AU, i.e., >10% of reference HIT serum. For A) AMA-1, (B) MSP-1_19_, (C) MSP-3, (D) CSP and (E) GLURP-R0, the proportion of children with no detectable parasitaemia (TS-PCR-), sub-microscopic (TS-PCR+) or microscopic (TS + PCR+) parasitaemia in each responder group is shown. Data were analysed by logistic regression and is shown for TS+ and all PCR+ individuals; values were adjusted for age. Additional file 10:
**Comparison of antibody titers between asymptomatic and symptomatic individuals in the end of the previous transmission season.** Humoral responses in the end of the previous transmission season (December 2011) were assessed by ELISA against (A) AMA-1, (B) MSP-1_19_, (C) MSP-3, (D) CSP and (E) GLURP-R0 for exposed children remaining asymptomatic (n = 47) or becoming symptomatic (n = 44) during the season. Reactivity for each antigen in undiluted hyperimmune Tanzanians (HIT) serum was set at 100 arbitrary units (AU). Differences between the two groups were analysed by linear regression of log-transformed (log10) data, adjusting values for age. Age adjusted P values are shown for each plot, with p-values without age adjustment (Mann–Whitney U test) in brackets. Scatter plots show individual data points, horizontal lines indicate the median of the group and error bars the interquartile range (IQR). Additional file 11:
**Comparison of antibody titers between asymptomatic and symptomatic individuals in the middle of the transmission season. **Humoral responses in the middle of the transmission season (September 2012) were assessed by ELISA against (A) AMA-1, (B) MSP-1_19_, (C) MSP-3, (D) CSP and (E) GLURP-R0 for exposed children remaining asymptomatic (n = 47) or becoming symptomatic (n = 44) during the season. Reactivity for each antigen in undiluted hyperimmune Tanzanians (HIT) serum was set at 100 arbitrary units (AU). Differences between the two groups were analysed by linear regression of log-transformed (log10) data, adjusting values for age. Age adjusted P values are shown for each plot, with p-values without age adjustment (Mann–Whitney U test) in brackets. Scatter plots show individual data points, horizontal lines indicate the median of the group and error bars the interquartile range (IQR). Additional file 12:
**Comparison of antibody titers between asymptomatic and symptomatic individuals after the transmission season.** Humoral responses were determined by ELISA after the end of transmission season (February 2013) in children who either did not experience any clinical episode of malaria during the transmission season (asymptomatic; n = 47), or had a symptomatic malaria episode at least once during the transmission season (symptomatic; n = 44). Antibody responses were determined for (A) AMA-1, (B) MSP-1, (C) MSP-3, (D) CSP and (E) GLURP-R0. Reactivity for each antigen in undiluted hyperimmune Tanzanians (HIT) serum was set at 100 arbitrary units (AU). Differences between the two groups were analysed by linear regression of log-transformed (log10) data, adjusting values for age. Age adjusted P values are shown for each plot, with p-values without age adjustment (Mann–Whitney U test) in brackets. Scatter plots show individual data points, horizontal lines indicate the median of the group and error bars the interquartile range (IQR).

## Abbreviations

AMA: Apical membrane protein
AU: Arbitrary unit
CSP: Circumsporozoite antigen
ELISA: Enzyme-linked immunosorbent assay
GLURP: Glutamine-rich protein
IQR: Interquartile range
MSP: Merozoite surface protein
OR: Odds ratio
