# Supplementary material for: Protection of Malian children from clinical malaria is associated with recognition of multiple antigens
Source: Malar J. 2015 Feb 5;14:56. doi: 10.1186/s12936-015-0567-9 (PMC4332451; doi:10.1186/s12936-015-0567-9)
Supplement: Additional file 1: — Primer sequences and product sizes. Provides details of primers used to detect Plasmodium falciparum DNA extracted from filter papers by PCR. [file 12936_2015_567_MOESM1_ESM.doc]

**Additional file 1: Primer sequences and product sizes**

|  | **Primer name** | **Sequences 5' - 3'** | **Length (bp)** | **Amplicon size (bp)** |
| --- | --- | --- | --- | --- |
| Nest 1 | rPLU6 | TTAAAATTGTTGCAGTTAAAACG | 23 | 1200 |
|  | rPLU5 | CYTGTTGTTGCCTTAAACTTC | 19 |  |
| Nest 2 | rFAL1 | TTAAACTGGTTTGGGAAAACCAAATATATT | 30 | 205 |
|  | rFAL2 | ACACAATAGACTCAATCATGACTACCCGTC | 30 |  |
