# Supplementary material for: Protection of Malian children from clinical malaria is associated with recognition of multiple antigens
Source: Malar J. 2015 Feb 5;14:56. doi: 10.1186/s12936-015-0567-9 (PMC4332451; doi:10.1186/s12936-015-0567-9)
Supplement: Additional file 2: — PCR Mastermix composition and PCR thermal cycler programme. Provides details of methodology and PCR cycling conditions used to detect Plasmodium falciparum DNA extracted from filter papers by PCR. [file 12936_2015_567_MOESM2_ESM.doc]

**Additional file 2: PCR Mastermix composition and PCR thermal cycler program**

| **Snounou N1 &N2** | | **N1&N2 cycler programme** | | |
| --- | --- | --- | --- | --- |
| PCR Buffer | 1x | 95°C | 5 min |  |
| MgCl2 | 2.5mM | 94°C | 60 sec | 35x |
| dNTPs | 0.2mM | 60°C | 60 sec |
| Primer | 0.25mM | 72°C | 90 sec |
| Polymerase | 2U | 72°C | 10 min |  |
