# Supplementary material for: Protection of Malian children from clinical malaria is associated with recognition of multiple antigens
Source: Malar J. 2015 Feb 5;14:56. doi: 10.1186/s12936-015-0567-9 (PMC4332451; doi:10.1186/s12936-015-0567-9)
Supplement: Additional file 3: — Demographic and parasitological parameters of total cohort. Provides demographic and parasitological data for all individuals that attended one or more of the four cross-sectional visits, out of which the longitudinal cohort of n = 99 children attending all visits was selected for immunological analysis. [file 12936_2015_567_MOESM3_ESM.doc]

**Additional file 3: Demographic and parasitological parameters of total cohort**

|  | Dec 2011 | Jul 2012 | Sept 2012 | Feb 2013 |
| --- | --- | --- | --- | --- |
| Number of individuals (N) attending each cross-sectional visit | n = 171 | n = 134 | n = 137 | n = 118 |
| Gender, % male (n/total) | 51.5% (88/107) | 52.2% (70/134) | 50.4% (69/137) | 56.8% (67/118) |
| Age in year, median (range) | 7 (2-14) | 7 (2-14) | 7 (2-14) | 7 (2-14) |
| Haemoglobin (g/dL), median (range) | 11.6 (7.2-14.5) | 11.1 (7.2-14.5) | 11.7 (6.5-14.6) | 12.2 (5.7-16.3) |
| Anaemia, % Hb <11 g/dL (n/total) | 24.6% (42/171) | 47.0% (63/134) | 27.2% (37/136) ^a^ | 16.1% (19/118) |
| Thick smear positive (%, n/total) | 19.3% (33/171) | 29.1% (39/134) | 21.9% (30/137) | 11.7% (13/111) |
| PCR positive (%, n/total) | n.d. | 79.4% (100/126)^a^ | 43.1% (56/130) ^a^ | n.d. |
| Parasitaemia in thick-smear positive individuals (n=35), median (range) | 11,400 (32-218,100) | 3,150 (100-132,275) | 925 (100-58,675) | 1725 (100-7,775) |

n.d. not done

a. for some children not all data were collected at the time of visit
