# Supplementary material for: Protection of Malian children from clinical malaria is associated with recognition of multiple antigens
Source: Malar J. 2015 Feb 5;14:56. doi: 10.1186/s12936-015-0567-9 (PMC4332451; doi:10.1186/s12936-015-0567-9)
Supplement: Additional file 4: — Correlation between age and humoral responses during follow-up. Spearman correlation analysis was performed to analyse for all n = 99 children the relationship between age and the height of antibody titers to the five malaria antigens AMA-1, MSP-119, MSP-3, CSP and GLURP-R0 at each of the four visits. [file 12936_2015_567_MOESM4_ESM.doc]

**Additional file 4:** Correlation between age and humoral responses during follow-up

| **Antigen** |  | **Dec 2011** | **Jul 2012** | **Sep 2012** | **Feb 2013** |
| --- | --- | --- | --- | --- | --- |
| **AMA-1** | Spearman r | 0,54 | 0,46 | 0,49 | 0,50 |
|  | 95% CI | 0,37 - 0,67 | 0,28 - 0,61 | 0,31 - 0,63 | 0,33 - 0,64 |
|  | ***P value*** | ***< 0,0001*** | ***< 0,0001*** | ***< 0,0001*** | ***< 0,0001*** |
| **MSP-1_19_** | Spearman r | -0,01 | 0,11 | 0,13 | -0,004 |
|  | 95% CI | -0,21 - 0,20 | -0,10 - 0,31 | -0,08 - 0,32 | -0,21 - 0,20 |
|  | ***P value*** | ***0,92*** | ***0,29*** | ***0,21*** | ***0,97*** |
| **MSP-3** | Spearman r | 0,36 | 0,22 | 0,31 | 0,28 |
|  | 95% CI | 0,17 - 0,53 | 0,02 - 0,41 | 0,12 - 0,49 | 0,08 - 0,45 |
|  | ***P value*** | ***0,0003*** | ***0,03*** | ***0,002*** | ***0,006*** |
| **CSP** | Spearman r | 0,45 | 0,27 | 0,41 | 0,47 |
|  | 95% CI | 0,27 - 0,60 | 0,067 - 0,45 | 0,22 - 0,56 | 0,29 - 0,61 |
|  | ***P value*** | ***< 0,0001*** | ***0,008*** | ***< 0,0001*** | ***< 0,0001*** |
| **GLURP-R0** | Spearman r | 0,49 | 0,38 | 0,43 | 0,52 |
|  | 95% CI | 0,32 - 0,63 | 0,19 - 0,54 | 0,25 - 0,58 | 0,36 - 0,66 |
|  | ***P value*** | ***< 0,0001*** | ***0,0001*** | ***< 0,0001*** | ***< 0,0001*** |
| **Number of Ags > 10AU** | Spearman r | 0,49 | 0,33 | 0,44 | 0,45 |
|  | 95% CI | 0,32 - 0,63 | 0,14 - 0,50 | 0,26 - 0,59 | 0,28 - 0,60 |
|  | ***P value*** | ***< 0,0001*** | ***0,0009*** | ***< 0,0001*** | ***< 0,0001*** |
