# Supplementary figures and images for: Protection of Malian children from clinical malaria is associated with recognition of multiple antigens
Source: Malar J. 2015 Feb 5;14:56. doi: 10.1186/s12936-015-0567-9 (PMC4332451; doi:10.1186/s12936-015-0567-9)

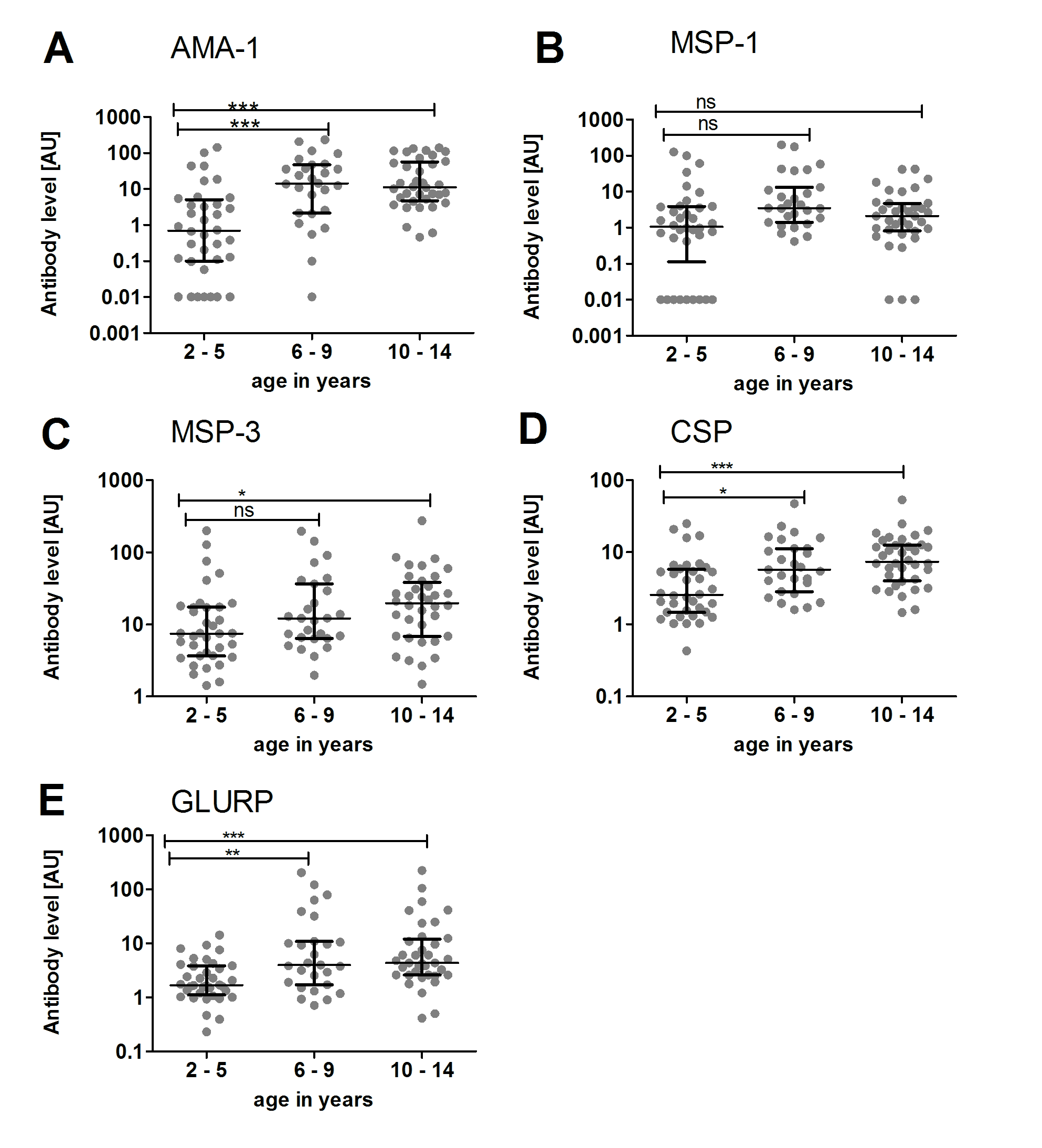

Supplement: Additional file 5: — Humoral responses by age in the middle of the transmission season. Antibody reactivity against P. falciparum antigens was tested by ELISA on samples from (n = 99) children collected in the middle of transmission (September 2012). A pool of sera from 100 hyperimmune Tanzanians (HIT) was used as a standard positive control. Reactivity for each antigen in undiluted HIT serum was set at 100 arbitrary units (AU). Humoral reactivity was assessed against (A) AMA-1, (B) MSP-119, (C) MSP-3, (D) CSP and (E) GLURP-R0. Responses between the three age groups were compared using Kruskal-Wallis with Dunn’s multiple comparison post-test. *p < 0.05; **p < 0.01; ***p < 0.001. Scatter plots show individual data points, horizontal lines indicate the median of the group and error bars the interquartile range (IQR). [file 12936_2015_567_MOESM5_ESM.tiff]

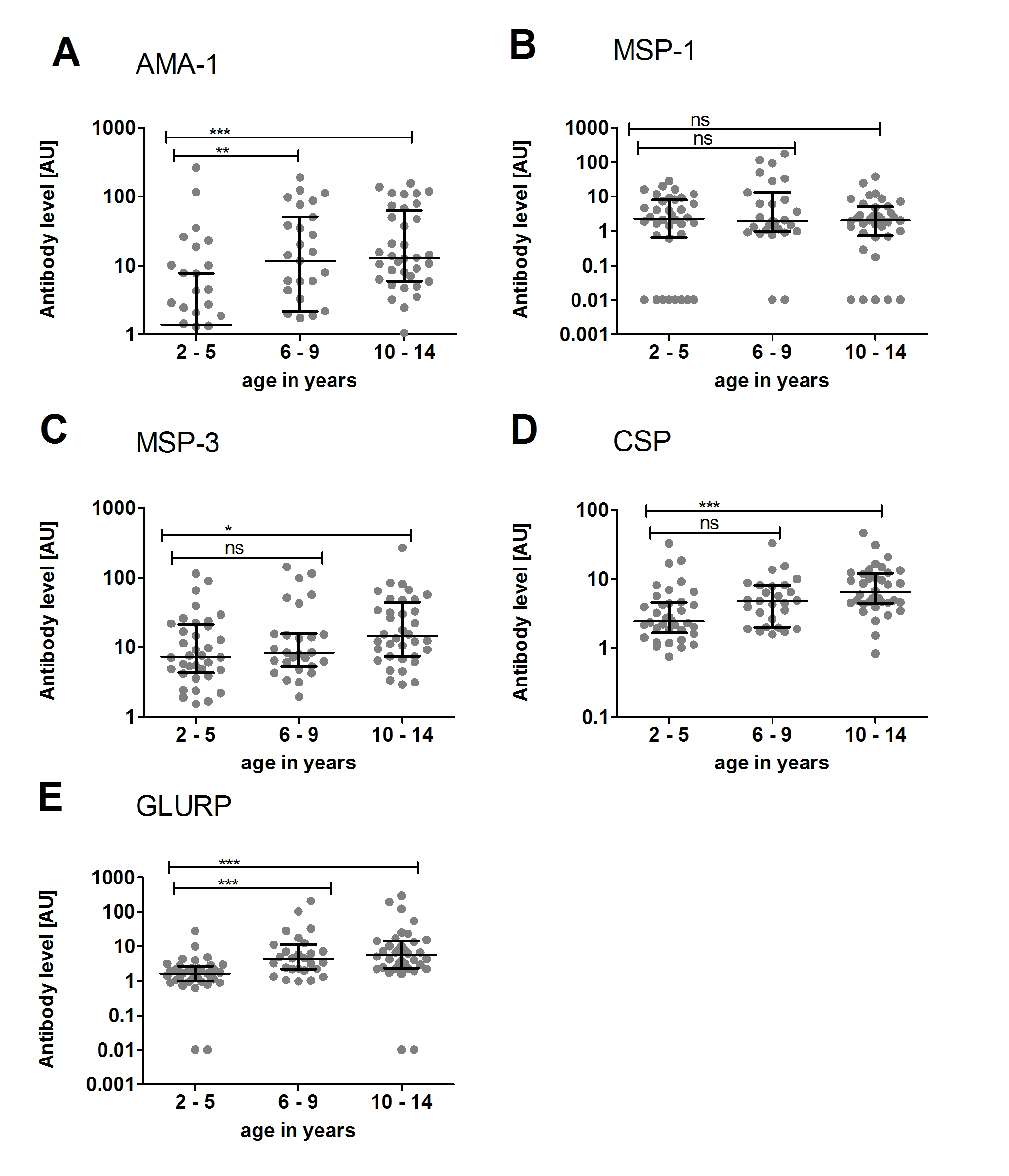

Supplement: Additional file 6: — Humoral responses by age after the transmission season. Antibody reactivity against P. falciparum antigens was tested by ELISA on samples from (n = 99) children collected after the transmission (February 2013). A pool of sera from 100 hyperimmune Tanzanians (HIT) was used as a standard positive control. Reactivity for each antigen in undiluted HIT serum was set at 100 arbitrary units (AU). Humoral reactivity was assessed against (A) AMA-1, (B) MSP-119, (C) MSP-3, (D) CSP and (E) GLURP-R0. Responses between the three age groups were compared using Kruskal-Wallis with Dunn’s multiple comparison post-test. *p < 0.05; **p < 0.01; ***p < 0.001. Scatter plots show individual data points, horizontal lines indicate the median of the group and error bars the interquartile range (IQR). [file 12936_2015_567_MOESM6_ESM.tiff]

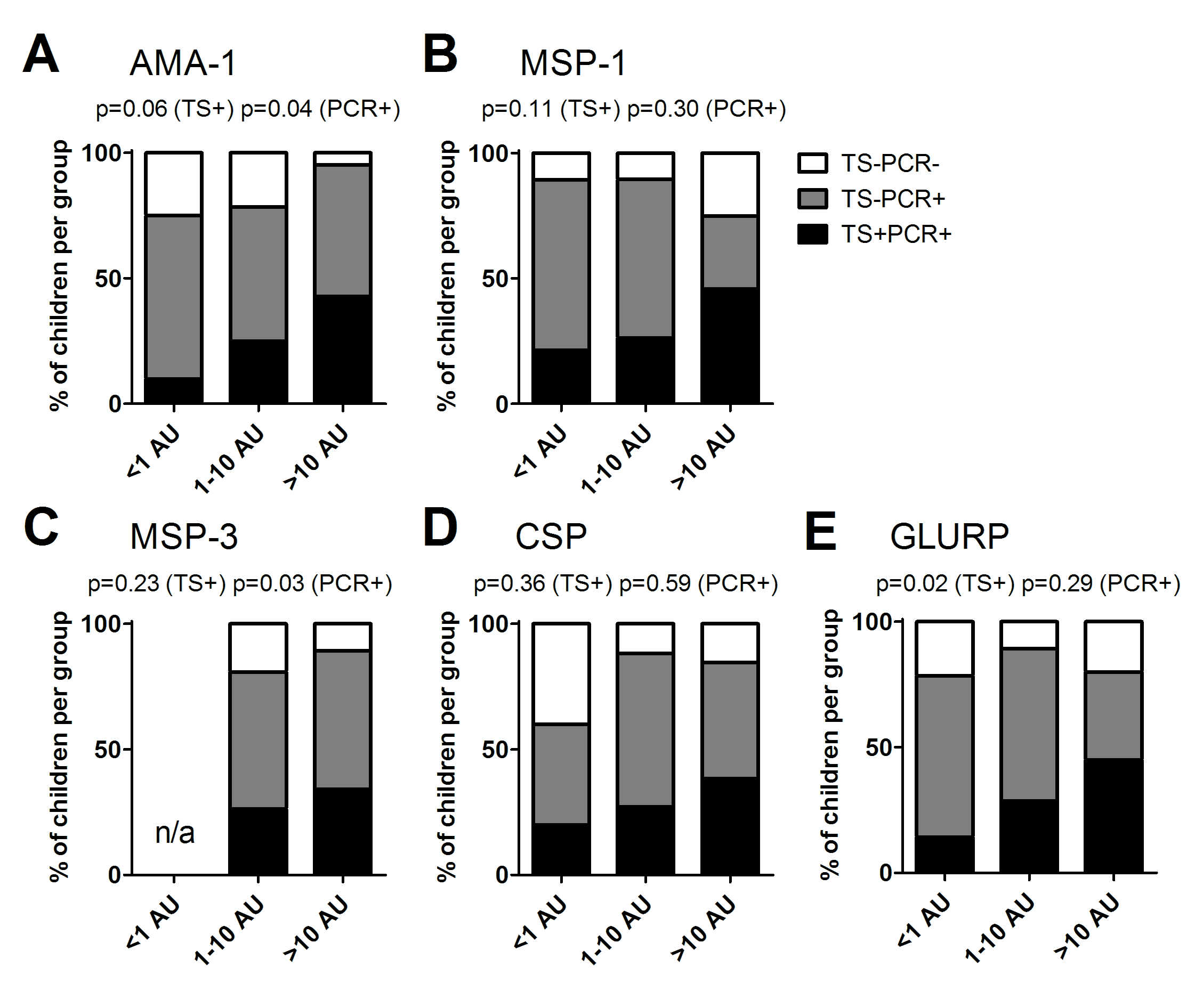

Supplement: Additional file 9: — Degree of parasitaemia in groups with different humoral reactivity. For each antigen, children were stratified into three groups depending on their response to this respective antigen early in the transmission season (July 2012) as follows: <1 AU, i.e., <1% of reference HIT serum; 1–10 AU; >10 AU, i.e., >10% of reference HIT serum. For A) AMA-1, (B) MSP-119, (C) MSP-3, (D) CSP and (E) GLURP-R0, the proportion of children with no detectable parasitaemia (TS-PCR-), sub-microscopic (TS-PCR+) or microscopic (TS + PCR+) parasitaemia in each responder group is shown. Data were analysed by logistic regression and is shown for TS+ and all PCR+ individuals; values were adjusted for age. [file 12936_2015_567_MOESM9_ESM.tiff]

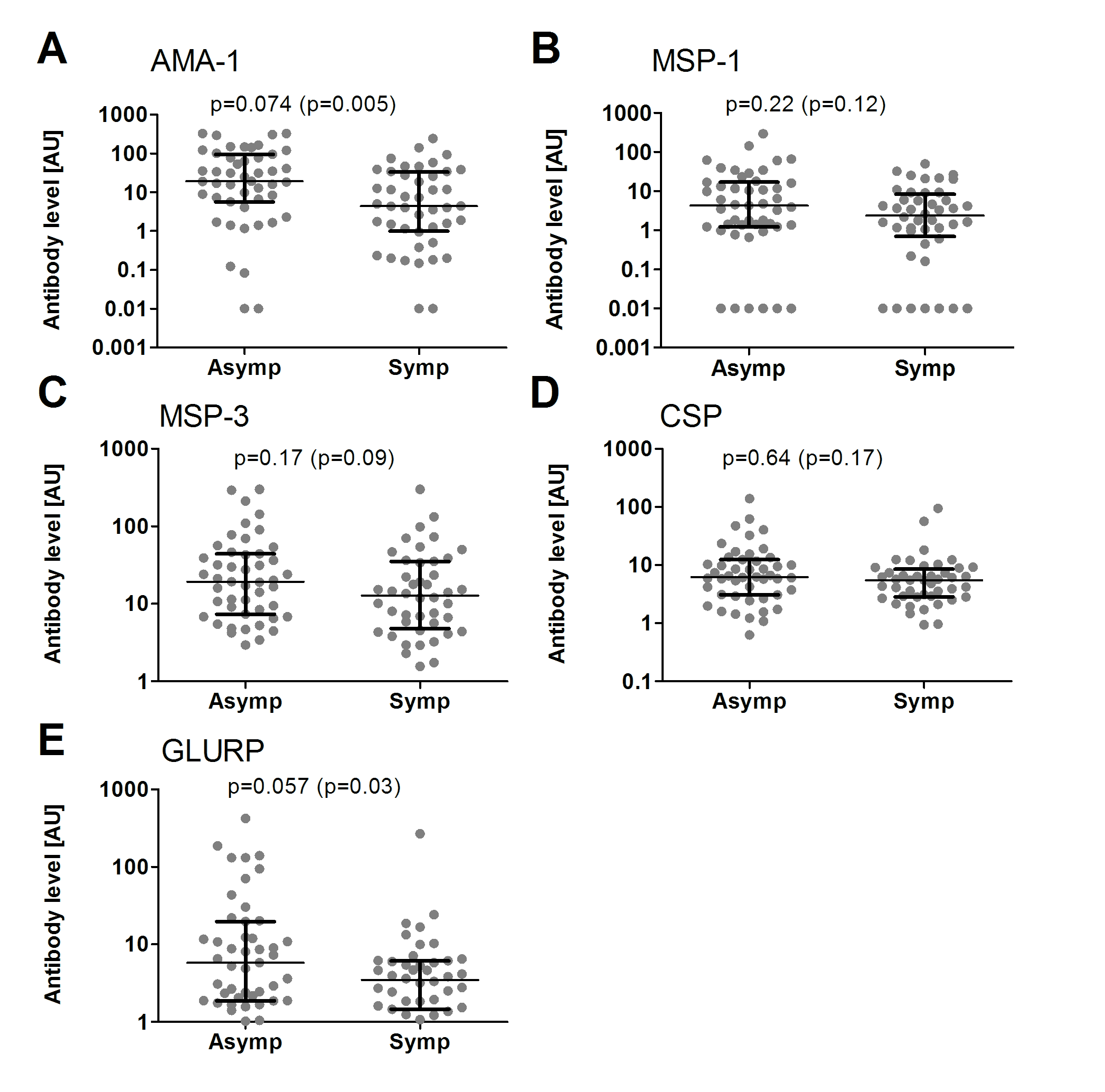

Supplement: Additional file 10: — Comparison of antibody titers between asymptomatic and symptomatic individuals in the end of the previous transmission season. Humoral responses in the end of the previous transmission season (December 2011) were assessed by ELISA against (A) AMA-1, (B) MSP-119, (C) MSP-3, (D) CSP and (E) GLURP-R0 for exposed children remaining asymptomatic (n = 47) or becoming symptomatic (n = 44) during the season. Reactivity for each antigen in undiluted hyperimmune Tanzanians (HIT) serum was set at 100 arbitrary units (AU). Differences between the two groups were analysed by linear regression of log-transformed (log10) data, adjusting values for age. Age adjusted P values are shown for each plot, with p-values without age adjustment (Mann–Whitney U test) in brackets. Scatter plots show individual data points, horizontal lines indicate the median of the group and error bars the interquartile range (IQR). [file 12936_2015_567_MOESM10_ESM.tiff]

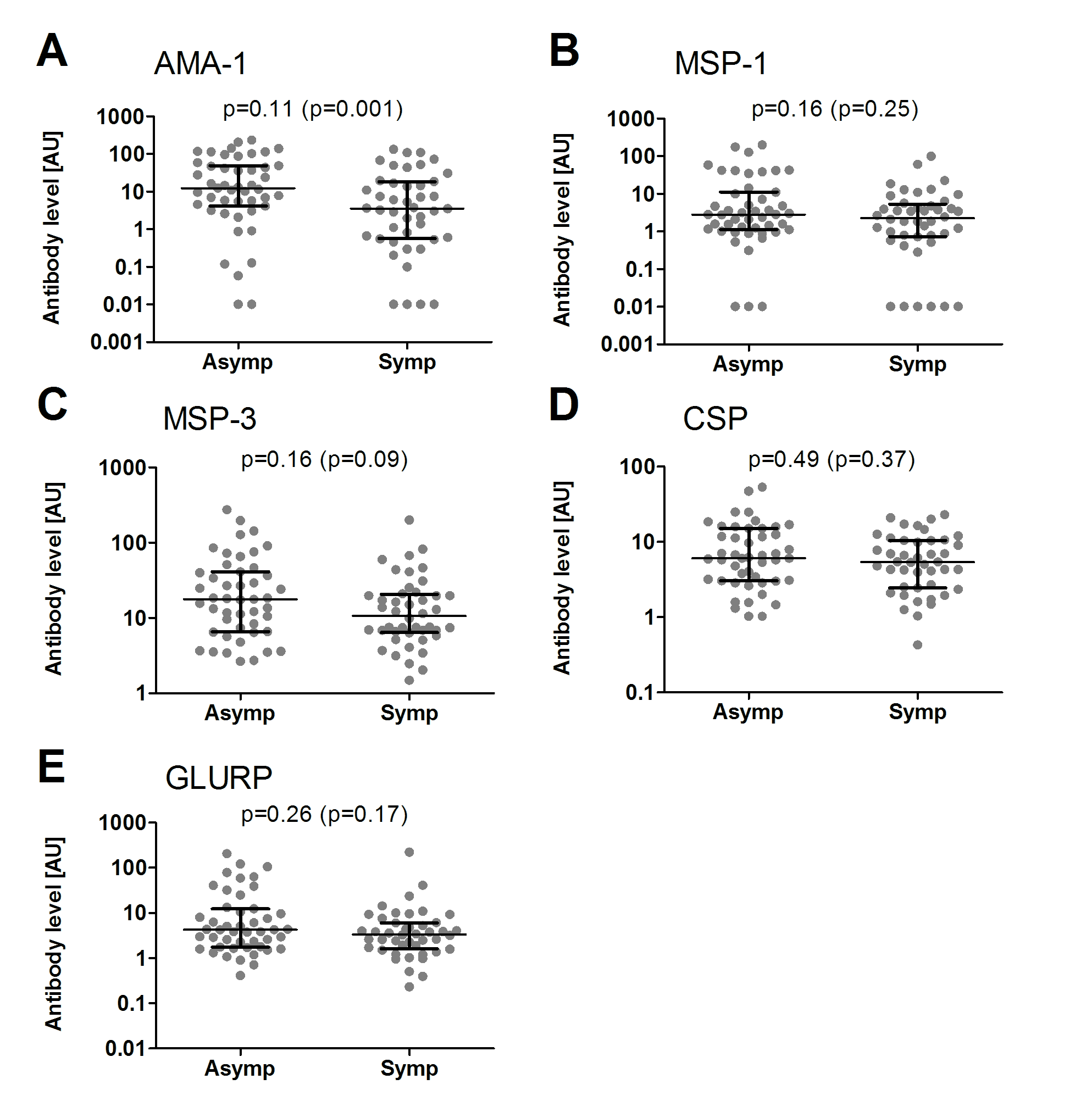

Supplement: Additional file 11: — Comparison of antibody titers between asymptomatic and symptomatic individuals in the middle of the transmission season. Humoral responses in the middle of the transmission season (September 2012) were assessed by ELISA against (A) AMA-1, (B) MSP-119, (C) MSP-3, (D) CSP and (E) GLURP-R0 for exposed children remaining asymptomatic (n = 47) or becoming symptomatic (n = 44) during the season. Reactivity for each antigen in undiluted hyperimmune Tanzanians (HIT) serum was set at 100 arbitrary units (AU). Differences between the two groups were analysed by linear regression of log-transformed (log10) data, adjusting values for age. Age adjusted P values are shown for each plot, with p-values without age adjustment (Mann–Whitney U test) in brackets. Scatter plots show individual data points, horizontal lines indicate the median of the group and error bars the interquartile range (IQR). [file 12936_2015_567_MOESM11_ESM.tiff]

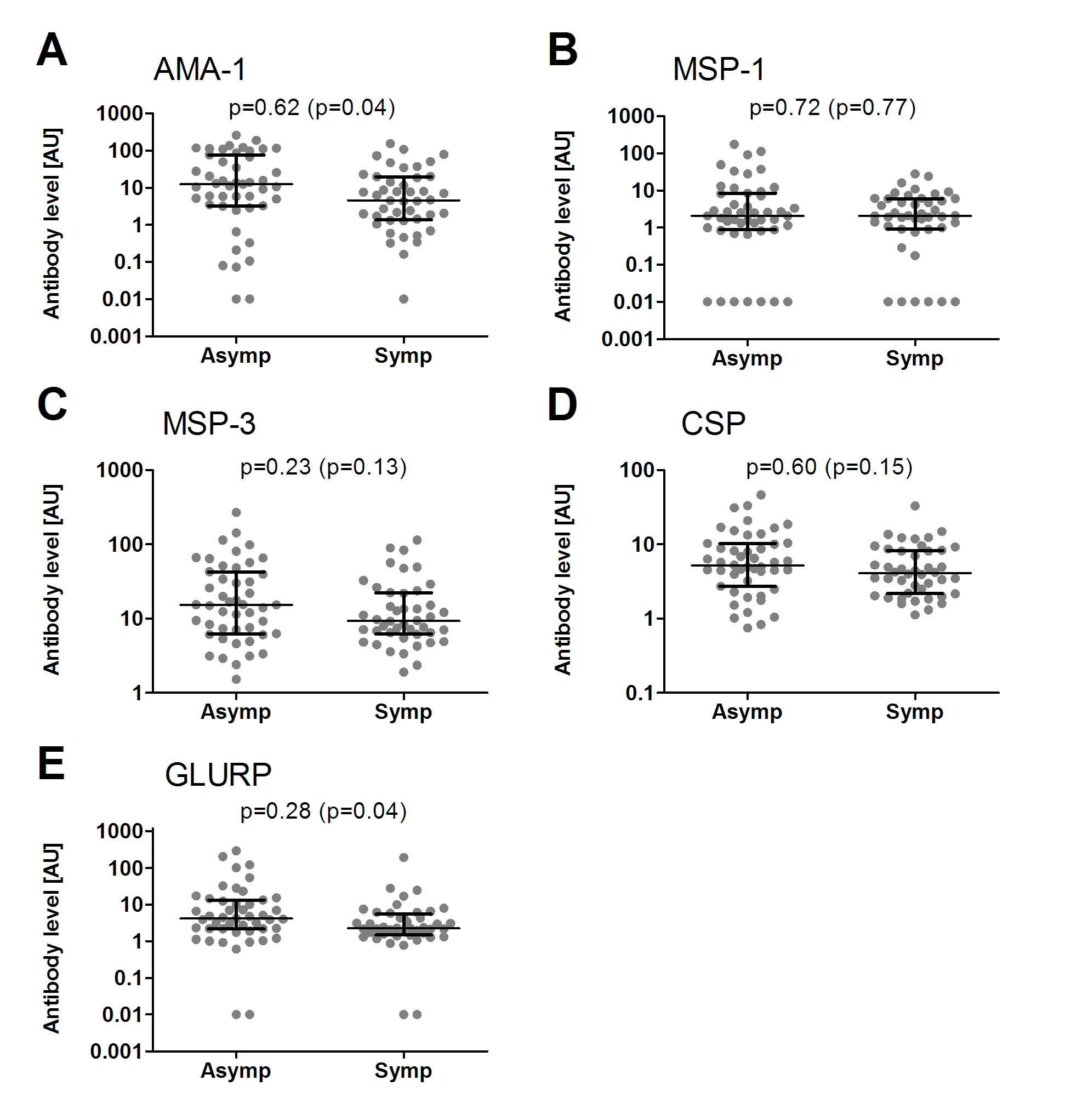

Supplement: Additional file 12: — Comparison of antibody titers between asymptomatic and symptomatic individuals after the transmission season. Humoral responses were determined by ELISA after the end of transmission season (February 2013) in children who either did not experience any clinical episode of malaria during the transmission season (asymptomatic; n = 47), or had a symptomatic malaria episode at least once during the transmission season (symptomatic; n = 44). Antibody responses were determined for (A) AMA-1, (B) MSP-1, (C) MSP-3, (D) CSP and (E) GLURP-R0. Reactivity for each antigen in undiluted hyperimmune Tanzanians (HIT) serum was set at 100 arbitrary units (AU). Differences between the two groups were analysed by linear regression of log-transformed (log10) data, adjusting values for age. Age adjusted P values are shown for each plot, with p-values without age adjustment (Mann–Whitney U test) in brackets. Scatter plots show individual data points, horizontal lines indicate the median of the group and error bars the interquartile range (IQR). [file 12936_2015_567_MOESM12_ESM.tiff]
