# Supplementary material for: Protection of Malian children from clinical malaria is associated with recognition of multiple antigens
Source: Malar J. 2015 Feb 5;14:56. doi: 10.1186/s12936-015-0567-9 (PMC4332451; doi:10.1186/s12936-015-0567-9)
Supplement: Additional file 7: — Changes of antibody titers after parasite exposure during the transmission season dependent on age. All n = 91 children that were exposed to the malaria parasite during follow-up were divided into age categories of two to five years (n = 35), six to nine years (n = 27) and ten to 14 years (n = 36). Antibody titers early during (July 2012) and after the transmission season (February 2013) were compared by Wilcoxon matched-pairs signed rank test. [file 12936_2015_567_MOESM7_ESM.doc]

**Additional file 7: Changes of antibody titers after parasite exposure during the transmission season dependent on age**

|  | **AMA-1** | **MSP-1_19_** | **MSP-3** | **CSP** | **GLURP-R0** |
| --- | --- | --- | --- | --- | --- |
| **2-5 years old** | n = 30^a^ | n = 30 | n = 30 | n = 30 | n = 30 |
| July 2012 median [IQR] | 1.25 [0.06-12.0] | 2.60 [0.57-9.6] | 7.6 [4.8-19.7] | 4.5 [1.3-10.1] | 2.4 [1.1-4.9 ] |
| Feb 2013 median [IQR] | 1.66 [0.31-7.7] | 2.28 [0.80-6.4] | 9.4 [4.9-22.7] | 2.6 [1.8-4.8] | 1.7 [1.1-2.8] |
| ***p-value ^b^*** | ***0.78*** | ***0.42*** | ***0.78*** | ***0.23*** | ***0.26*** |
|  |  |  |  |  |  |
| **4-9 years old** | n = 25^a^ | n = 25 | n = 25 | n =25 | n = 25 |
| July 2012 median [IQR] | 11.4 [1.6-52.8] | 2.3 [0.6-21.8] | 13.1 [5.1-30.3] | 6.1 [1.9-11.0] | 4.0 [0.9-13.8] |
| Feb 2013 median [IQR] | 11.7 [2.7-63.1] | 2.1 [1.0-20.6] | 8.4 [6.2-29.1] | 4.9 [2.6-8.1] | 4.5 [2.2-9.7] |
| ***p-value ^b^*** | ***0.06*** | ***0.64*** | ***0.43*** | ***0.35*** | ***0.96*** |
|  |  |  |  |  |  |
| **10-14 years old** | n = 36^a^ | n = 36 | n = 36 | n = 36q | n = 36 |
| July 2012 median [IQR] | 14.2 [3.4-56.3] | 2.1 [0.7-7.6] | 16.1 [4.8-62.9] | 6.7 [4.2-11.6] | 4.8 [2.6-12.5] |
| Feb 2013 median [IQR] | 12.8 [5.9-62.7] | 2.0 [0.7-5.1] | 14.4 [7.4-44.1] | 6.4 [4.5-12.1] | 5.6 [2.3-14.2] |
| ***p-value ^b^*** | ***0.33*** | ***0.49*** | ***0.19*** | ***0.88*** | ***0.22*** |

a. 91 children that experiences parasitemia detected by qPCR or thick smear where included in this analysis

b. Antibody reactivity was compared between July and February for each group and antigen by Wilcoxon matched-pairs signed rank test
