# Supplementary material for: Protection of Malian children from clinical malaria is associated with recognition of multiple antigens
Source: Malar J. 2015 Feb 5;14:56. doi: 10.1186/s12936-015-0567-9 (PMC4332451; doi:10.1186/s12936-015-0567-9)
Supplement: Additional file 8: — Frequency of clinical malaria episodes in children stratified based on their reactivity to different Plasmodium falciparum antigens. All n = 91 children that were exposed to the malaria parasite during follow-up were stratified based on their antibody responses to individual malaria antigens in July 2012 (early in the transmission season) as follows: <1 AU, i.e., <1% of reference HIT serum; 1–10 AU; >10 AU, i.e., >10% of reference HIT serum. Additionally children were also stratified based on the number of antigens strongly recognized at this time point, using an arbitrary cut-off of 10 AU (0 antigens, 1–2 antigens and 3–5 antigens). The proportion of children developing clinical malaria in each group during follow-up is shown. [file 12936_2015_567_MOESM8_ESM.doc]

**Additional file 8: Frequency of clinical malaria episodes in children stratified based on their reactivity to different Pf antigens**

| **Antigen** | **Antibody reactivity (AU)** | **Clinical malaria during follow-up** | |
| --- | --- | --- | --- |
|  |  | n/total | % |
| **AMA-1** | < 1AU | 14/21 | 66.7% |
|  | 1-10 AU | 16/28 | 57.1% |
|  | > 10 AU | 14/42 | 33.3% |
| **MSP-1_19_** | < 1AU | 15/28 | 53.6% |
|  | 1-10 AU | 18/39 | 46.2% |
|  | > 10 AU | 11/24 | 45.8% |
| **MSP-3** | < 1AU | 1/1 | 100.0% |
|  | 1-10 AU | 24/43 | 55.8% |
|  | > 10 AU | 19/47 | 40.4% |
| **CSP** | < 1AU | 4/6 | 66.7% |
|  | 1-10 AU | 28/59 | 47.5% |
|  | > 10 AU | 12/26 | 46.2% |
| **GLURP-R0** | < 1AU | 9/15 | 60.0% |
|  | 1-10 AU | 28/56 | 50.0% |
|  | > 10 AU | 7/20 | 35.0% |
| **Multiple Ags** | no Ag > 10AU | 15/24 | 62.5% |
|  | 1-2 Ags > 10AU | 18/43 | 41.9% |
|  | 3-5 Ags > 10AU | 10/24 | 41.7% |
